# Structural, elastic, electronic, optical and anisotropy properties of newly quaternary Tl2HgGeSe4 via DFPT predictions associated to XPES and RS experiments

**DOI:** 10.1038/s41598-024-67231-2

**Published:** 2024-07-15

**Authors:** Mohamed Salah Halati, Oleg Yu Khyzhun, Abderrazak Khireddine, Michal Piasecki, Ilona Radkowska, Khaled Hamdi Cherif, Zakia Lounis, Yves Caudano, Abdelhak Bedjaoui, Ahmad Alghamdi, Prabhu Paramasivam, Chander Prakash, Sherif S. M. Ghoneim

**Affiliations:** 1 Scientific and Technical Research Centre for Arid Areas (C.R.S.T.R.A), water resources and treatments team, Omar El Barnaoui, Campus of Mohamed, Khider University of Biskra, 07000 Biskra, Algeria; 2 Laboratory of Materials (LabMat), National Polytechnique School (ENP-Maurice Audin -Oran-), El M’naouer, P.O. Box 1523, 31000 Oran, Algeria; 3 Modeling and simulations for Laser applications team, Research Center in Industrial Technologies (CRTI), P. O. Box 64, 16014 Cheraga, Algiers Algeria; 4 Frantsevych Institute for Problems of Materials Science, National Academy of Sciences of Ukraine, 3 Krzhyzhanivsky Street, Kyiv, 03142 Ukraine; 5 Department of Experimental Physics and Information-Measuring Technology, Lesya Ukrainka Volyn National University, 13 Voli Avenue, Lutsk, UA-43025 Ukraine; 6 Laboratory for Developing New Materials and Their Characterizations, Department of Physics, Faculty of Science, University Ferhat Abbas Setif 1, 19000 Setif, Algeria; 7 Jan Dlugosz University in Częstochowa, Armii Krajowej 13/15, PL-42-217 Częstochowa, Poland; 8 Research Unit Lasers and Spectroscopies (UR-LLS), naXys & NISM, Université de Namur, Rue de Bruxelles 61, 5000 Namur, Belgium; 9 Department of Technology, Faculty of Technology, Bejaia University, 6000 Bejaia, Algeria; 10 Department of Mechanical and Industrial Engineering, College of Engineering and Computing in Al-Qunfudhah, Umm Al-Qura University, Mecca, Saudi Arabia; 11 Centre of Research Impact and Outcome, Chitkara University, Rajpura, Punjab 140401 India; 12 Department of Mechanical Engineering, Mattu University, 318 Mettu, Ethiopia; 13 University Centre for Research and Development, Chandigarh University, Mohali, Punjab 140413 India; 14 Department of Electrical Engineering, College of Engineering, Taif University, P.O. BOX 11099, 21944 Taif, Saudi Arabia

**Keywords:** Ge L_2_M_45_M_45_, VB-XPES, Raman spectroscopy, DFPT calculation, Nanoscale, Electronic properties and materials, Thermodynamics, Structural materials

## Abstract

In the present work, we report on theoretical studies of thermodynamic properties, structural and dynamic stabilities, dependence of unit-cell parameters and elastic constants upon hydrostatic pressure, charge carrier effective masses, electronic and optical properties, contributions of interband transitions in the Brillouin zone of the novel Tl_2_HgGeSe_4_ crystal. The theoretical calculations within the framework of the density-functional perturbation theory (DFPT) are carried out employing different approaches to gain the best correspondence to the experimental data. The present theoretical data indicate the dynamical stability of the title crystal and they reveal that, under hydrostatic pressure, it is much more compressible along the a-axis than along the c-axis. Strikingly, the charge effective mass values ($m_{e}^{{^{*} }}$ and $m_{h}^{{^{*} }}$) vary considerably when the high symmetry direction changes indicating a relative anisotropy of the charge-carrier’s mobility. Furthermore, the Young modulus and compressibility are characterized by the maximum and minimum values ($E^{max}$ and $E^{\min }$) and ($\beta^{max}$ and $\beta^{\min }$) that are equal to (62.032 and 28.812) GPa and (13.672 and 6.7175) TPa^–1^, respectively. Additionally, we have performed calculations of the Raman spectra (RS) and reached a good correspondence with the experimental RS spectra of the Tl_2_HgGeSe_4_ crystal. The XPES associated to RS constitutes powerful techniques to explore the oxidized states of Se and Ge in Tl_2_HgGeSe_4_ system.

## Introduction

Quaternary Tl_2_HgGeSe_4_ crystal that was recently synthesized in Ref.^1^ belongs to an interesting family of chalgogenides crystallizing in orthorhombic (*Cmc*2_1_ and *Pmn*2_1_) and tetragonal (*I* $\overline{4}$ and *I* $\overline{4}$ 2m) space groups with a common formula A_2_B^II^D^IV^Q_4_ (A stands for Cu/Ag, B^II^ is referred to Zn/Cd/Hg, D^IV^ serves for Si/Ge/Sn, whereas Q is S/Se/Te) that are of tremendous attention over recent decades from theoretical and technological viewpoints because of a number of practical advantages. Among them are p-type electrical conductivity, energy band gaps being in the range of 1.0–1.6 eV, high values of conversion power and absorption coefficients over 10^4^ cm^–1^^2–6^ that make these compounds to be indispensable photovoltaic absorbers for novel technologies of thin-film production for solar-cells^7,8^, photocatalysts of different conversion reactions ^9^, upcoming thermoelectric and hole transport semiconductors^10,11^, prospective materials for nonlinear optical (NLO) applications^12^. The tremendous challenge for scientists and engineers over recent decade is to tune the physicochemical properties of the A_2_B^II^D^IV^Q_4_ chalcogenides to gain unambiguous technological values by chemical alloying, formation of point defects and secondary/metastable phases, preparing solid-solutions^13,14^, changing crystal dimensions to nanoscales, etc.^15^.

It is well known that thallium belongs to the 13th group of the periodic table and it is characterized by three valence electrons on the outer shells (6s^2^ and 6p^1^). However, the inert Tl 6s^2^ electronic pairs are characteristic for this chemical element, whereas copper belongs to the 11th group of the periodic table possessing one 4s electron on top of the filled d shells. These peculiarities of the above mentioned chemical elements are the main reason that, in many cases, thallium behaves like copper, in particular, it can substitute copper in the Cu_2_B^II^D^IV^Q_4_ chalcogenides^16^. Nevertheless, unlike very broad family of Cu_2_B^II^D^IV^Q_4_ chalcogenides^17^, the thallium bearing Tl_2_B^II^D^IV^Q_4_ family is quite limited. In particular, at present the existence of quaternary tellurides Tl_2_AD^IV^Te_4_ (where A = Cd, Hg, Mn, while D^IV^ stands for Ge and Sn)^18^ and Tl_2_B^II^SiTe_4_ (B^II^ = Cd, Hg)^19^, selenides Tl_2_HgD^IV^Se_4_ (D^IV^ = Sn, Ge)^1,20,21^ and Tl_2_CdD^IV^Se_4_ (D^IV^ = Sn, Ge)^19^, as well as sulfide Tl_2_HgSnS_4_^22^, has been confirmed. All the above-mentioned Tl_2_B^II^D^IV^Q_4_ chalcogenides crystallize in the isostructural family within a noncentrosymmetric tetragonal *I* $\overline{4}$ 2*m* space group; therefore, these compounds attract attention as very promising NLO materials^1^.

Among the Tl_2_B^II^D^IV^Q_4_ chalcogenides, thallium mercury germanium selenide, Tl_2_HgGeSe_4_, is of particular interest. Recent studies of the Tl_2_HgGeSe_4_ crystal^1^ indicate that this compound belongs to a family of photosensitive non-direct p-type semiconductors and the crystal reveals low moisture sensitivity being sensitive to Ar^+^ ion-beam-induced-bombardment. Further, Tl_2_HgGeSe_4_ compound is characterized by a rather substantial covalent component of the chemical bonding and this covalent component is provided through high hybridization degree of Se 4p states with Hg(Tl) 6p states in the upper part of the valence band, with Ge 4p states in its central portion, and with Hg(Tl) 6s states in the lower part of the band. In the latter work, it was established that the main peculiarities of the occupation of the valence-band region of Tl_2_HgGeSe_4_ resemble those of its Cd- and Sn-bearing counterparts Tl_2_CdD^IV^Se_4_ (D^IV^ = Sn, Ge)^23–25^ and Tl_2_HgSnSe_4_^26^. Furthermore, the Raman spectra of the Tl_2_HgGeSe_4_ crystal measured employing two laser excitations, 532 nm and 830 nm, reveal a number of vibration modes that could be associated with chemical bonds related to the nearest neighbors of the atoms composing the studied crystal^1^.

However, due to a comparative novelty of the Tl_2_HgGeSe_4_ crystal, its thermodynamic properties, structural and dynamic stabilities, dependence of unit-cell parameters and elastic constants upon hydrostatic pressure, charge carrier effective masses, contributions of interband transitions in the 1st Brillouin zone, theoretical verifications of the experimental X-ray PhotoElectron spectroscopy (XPES) features and Raman spectra of the crystal under consideration have not been performed in Ref.^1^. To fill this lack, in this study we employ different theoretical functional (GGA-PBEsol08, TB-mBJ and new KTB-mBJ) aiming to explore the structural features, thermodynamic stability, phonon DOS curves, the impact of hydrostatic pressure at both lattice parameters and elastic constants. We aim also to give insights at the electronic band dispersions using various exchange and correlation potentials. We focus also to evaluate the charge carrier effective masses toward different high symmetry points in the BZ. Based on the shape of the valence-band XPES spectra, an adequate clarification was given to describe the state of the shallow layers of the title compound. The present theoretical calculations of Raman spectra are very helpful to confirm experiments.

## Theoretical details: methods and settings

All the current results derived in the whole of theoretical investigations which include the lattice parameters (a and c), atomic position coordinates, phonons and the associated density of states (DOS) and Raman spectra of the Tl_2_HgGeSe_4_ crystal are obtained using the pseudo potential plane-wave (PP-PW) technique as realized in the CASTEP code^27^, because the implemented generalized gradient approximation of Perdew-Burke-Ernzerhof for solids GGA‒PBEsol08^28,29^ is considered as an accurate exchange–correlation energy which is more susceptible to obtain lattice parameters that agree well with the experimental counterparts^30,31^. The electronic properties and optical parameters were carried out by means of the full potential linearized augmented plane wave plus local orbitals (FP-LAPW + lo) method^32^ as embedded in the WIEN2k code. These methods are within the framework of the density-functional Perturbation Theory (DFPT)^33^. The so-called Vanderbilt-type ultra soft pseudo potential (US-PP)^34^ was also used to comprise the potential presented by the valence electrons regarding the frozen core electrons and the nuclei.

Data listed in Table 1 show the valence electron states of Tl, Hg, Ge and Se atoms which are considered explicitly treated. The geometric optimizations were determined using the Broyden–Fletcher–Goldfarb–Shanno (BFGS) minimization technique^35^, which gives a proper way to obtain the lowest energy structure. The hydrostatic pressure coupled with the variable cell approach is applied to perform a full optimization of the cell parameters and elastic constants (C_ij_)_(s)_ toward each target of the external pressure. Besides, in order to calculate the Phonon dispersion spectra and phonon DOS, we employ the finite displacement method performed at the Tl_2_HgGeSe_4_ primitive unit cell. The employing convergence plane wave energy cutoff that was set to be equal to 500 eV, and the Monkhorst–Pack grids for the sampling generate a Brillouin zone of $\frac{\pi }{10}$ Å^–1^. Further, the use of the FP-LAPW + lo method is considered among the most suitable approaches to acquire an accurate electronic structure and optical parameters of solids. A long time ago, many theoretical attempts in this field occurred as consecutive constructed potentials used instead of the conventional GGA to remedy the remarkable underestimation compared to the experimental optical band gap. So, in this work, we adopt in the establishment of electronic band structure the implementation of the Tran-Blaha modified semi-local Becke-Johnson exchange potential (TB-mBJ)^36^ and another potential recently re-parameterized by Koller, Tran and Blaha (which is denoted new KTB‒mBJ)^36,37^. These potentials are efficient points of view to reproduce accurate optical band gaps for quite a number of semiconductors and insulators being in fair agreement with experiments compared to others by means of the high-cost hybrid functional and GW methods^38^. In order to comprehend more the relativistic effect at the deep core electrons of the heavy elements such as Tl and Hg, we have evaluated the examined properties without and with the spin–orbit coupling effect (WSOC/SOC). The later approach gives a rise to better estimation of the main feature of the titled compound. The unit cell is divided into non-overlapping spheres labeled muffin-tin spheres (MTS)(s) and between each MTS(s) there is a space called the interstitial region (IR). Linear combinations of radial atomic functions and their energy derivatives times spherical harmonics are used to expand the wave functions inside the MTS, whereas a plane wave basis set is used in the IR. The cut-off K_max_ was chosen equal to 10/${R}_{MT}^{min}$ (${R}_{MT}^{min}$ K_max_ = 10, ${R}_{MT}^{min}$ is the smallest muffin-tin sphere radius). We chose enough values of MTS equal to 2.50 Bohr for Tl, 2.39 Bohr for Hg, 2.14 Bohr for Ge and 2.14 Bohr for Se, which are appropriate to ensure no charge leakage out of the MTS. The irreducible wedge in the Brillouin zone integration was replaced by a summation over 10 × 10 × 8 Monkhorst–Pack^39^ k-points. The self-consistent field iterations were repeated until the calculated total energy of the crystal converged to less than 10^5^ Ry and the maximum force on any atom was smaller than 0.1 eV/Å.

**Table 1:** The electronic configurations of atoms constituting Tl_2_HgGeSe_4_.

Tl_2_HgGeSe_4_				
Atom	Z	Core electrons	Semi-core electrons	Valence electrons
Tl (Thallium)	81	1s^2^ 2s^2^ 2p^6^ 3s^2^ 3p^6^ 3d^10^ 4s^2^ 4p^6^ 4d^10^ 4f^14^ 5s^2^	5p^6^ 5d^10^	6s^2^ 6p^1^
Hg (Mercury)	80	1s^2^ 2s^2^ 2p^6^ 3s^2^ 3p^6^ 3d^10^ 4s^2^ 4p^6^ 4d^10^ 4f^14^ 5s^2^	5p^6^ 5d^10^	6s^2^
Ge (Germanium)	32	1s^2^ 2s^2^ 2p^6^ 3s^2^ 3p^6^	3d^10^	4s^2^ 4p^2^
Se (Selenium)	34	1s^2^ 2s^2^ 2p^6^ 3s^2^ 3p^6^	3d^10^	4s^2^ 4p^4^

Core, semi-core and valence electrons are well presented by the Kleshkowski rule.

## Experimental set up

In order to validate as possible our DFPT calculations, we use the Tl_2_HgGeSe_4_ single-phase crystal that was grown successfully and for the first time by Vu et al.^1^ via vertical Bridgman-Stockbarger (VBS) technology^40^ in which important steps were undertaken. The adequate conditions were also inspired by the analysis of the T-x phase diagram of the Tl_2_Se–HgSe–GeSe_2_ system^41,42^. The X-ray powder diffraction (XRPD) analysis data show that the crystal under study is a single-phase Tl_2_HgGeSe_4_ system (a = b = 7.9984(2) Å and c = 6.7645(2) Å) belonging to tetragonal space group I4̅2m (${D}_{2d}^{11}$) through Hermann-Mauguin (Schoenflies) notation^43,44^. The major colours that appear at the surface of the considered system are navy blue and light pink. This iridescence could be interpreted by the current DFPT results vide infra. Both X-ray Photo Electron Spectroscopy (XPES) and the in-situ Ar^+^ ion sputtering were performed with the UHV-Analysis-System elaborated by SPECS Surface Nano Analysis Company (Berlin, Germany) as reported in Ref.^1^. The sample was subjected to the UHV X-Ray excitation with 7.1 × 6.2 × 1.3 mm^3^ dimensions. The XPES spectra acquisition was made in the UHV-Analysis-System at residual pressure of about 10^–8^ mbar employing an X-ray excitation of a magnesium anode (Mg K $\alpha$ $h\nu$ = 1.2536 keV). The measured spectra were recorded at a constant pass energy of (28.00 $\pm$ 0.05) eV. Because of the hydrocarbons adsorbed on the upper layer of the system under study, the related peak C 1 s core level photoemission was adjusted to be equal to 284.6 eV as it is recommended for XPES analysis of Cu(Tl)_2_B^II^D^IV^X_4_ family^5,23,24,45^. It should be noted that very often there exists a superposition between the Auger line (Ge L_2_M_23_M_23_) with the C 1 s photoelectron line, in addition to the charging surface effect. In order to avoid the electrical field arising from the surface charge, an important UHV treatment was done such as in the case of germanium (Ge) bearing Cu(Tl)_2_B^II^D^IV^X_4_ chalcogenides^5,23,24^. We adopted in cleaning process the Ar^+^ ions sputtering under UHV condition with E = 3000 eV, a current density of about 13μA per cm^2^, during a time of 5 min and the entire Ar^+^ flux is equal to 5.3 × 10^16^ ions⋅cm^−2^.

Room temperature (RT) Raman spectroscopy measurements were performed using a Renishaw In Via 3RTG68 Spectrometer which was equipped with a highly sensitive Renishaw Centres 3CMC21 detector. The use of two different laser excitation wavelengths ${\lambda }_{exc,1}$= 532 nm and ${\lambda }_{exc,2}$= 830 nm was assured to cover the spectral range [48.65 to 4000.05 cm^–1^] and [94.6 to 4000.05 cm^–1^] of Raman shift, respectively.

## Results and discussion

### Structural, thermodynamic and dynamical stabilities

#### Structural and thermodynamic stabilities

Aiming to explore structural and thermodynamic stabilities of the quaternary Tl_2_HgGeSe_4_ compound, both cohesive energy ($E_{coh}$) and formation enthalpy (${\Delta }H_{For}$) have been evaluated. The first one is described as the energy required when a system decomposes into free atoms, employing the following relationship^46^:

$$ E_{coh} \left[ {\left( {Tl_{2} HgGeSe_{4} } \right)_{Tetra} } \right] = \frac{{E_{Tot}^{{Tl_{2} HgGeSe_{4(solid)}^{I - 42m} }} - \left( {N_{Tl} E_{Tot}^{{Tl\left( {atom} \right)}} + N_{Hg} E_{Tot}^{{Hg\left( {atom} \right)}} + N_{Ge} E_{Tot}^{{Ge\left( {atom} \right)}} + N_{Se} E_{Tot}^{{Se\left( {atom} \right)}} } \right)}}{{N_{Tl} + N_{Hg} + N_{Ge} + N_{Se} }}. $$

where $E_{Tot}^{{Tl_{2} HgGeSe_{4(solid)}^{I - 42m} }}$, $E_{Tot}^{{Tl\left( {atom} \right)}}$, $E_{Tot}^{{Hg\left( {atom} \right)}}$, $E_{Tot}^{{Ge\left( {atom} \right)}}$ and $E_{Tot}^{{Se\left( {atom} \right)}}$ refer to the total energy of the Tl_2_HgGeSe_4_ primitive cell and the total energies of the isolated Tl(Thallium), Hg(Mercury), Ge(Germanium) and Se(Selenium) atoms, respectively. We calculated the free energy of the above atoms considering a cubic box which has a large lattice constant (a $\sim 10$Å) that contains the considered atom. So, the predictive cohesive energy is equal to $- \, 4.872{\text{ eV}}{\text{.atom}}^{ - 1}$ using the so-called generalized gradient approximation functional of Perdew-Burke-Ernzerhof (GGA-PBEsol08). The second one was the formation enthalpy (${\Delta }H_{For}$) which is described as the energy needed to form a system from its constituent elements in their most stable state as following^46^:

$$ \Delta H_{For} \left[ {\left( {Tl_{2} HgGeSe_{4} } \right)_{Tetra} } \right] = \frac{{E_{Tot}^{{Tl_{2} HgGeSe_{4(solid)}^{I - 42m} }} - \left( {N_{Tl} E_{Tot}^{{Tl_{{\left( {solid} \right)}}^{fcc} }} + N_{Hg} E_{Tot}^{{Hg_{{\left( {solid} \right)}}^{bct} }} + N_{Ge} E_{Tot}^{{Ge_{{\left( {solid} \right)}}^{bcc} }} + N_{Se} E_{Tot}^{{Se_{{\left( {solid} \right)}}^{hcp} }} } \right)}}{{N_{Tl} + N_{Hg} + N_{Ge} + N_{Se} }}, $$

where $E_{Tot}^{{Tl_{{\left( {solid} \right)}}^{fcc} }}$, $E_{Tot}^{{Hg_{{\left( {solid} \right)}}^{bct} }}$, $E_{Tot}^{{Ge_{{\left( {solid} \right)}}^{bcc} }}$ and $E_{Tot}^{{Se_{{\left( {solid} \right)}}^{hcp} }}$ refer to the total energy of the “face-centered cubic Tl”, “body-centred tetragonal Hg”, “body-centred cubic Ge” and “hexagonal close packed Se” solids, respectively. The formation energy (${\Delta }H_{For}$) is equal to $- \, 1.384{\text{ eV}}{\text{.atom}}^{ - 1}$ as determined using the same exchange and correlation potential. The negative values of both ($E_{coh}$) and (${\Delta }H_{For}$), indicate the feasibility of the atomic agglomeration and the thermodynamic stability of the system under study.

#### Dynamical stability

**Figure 1:**
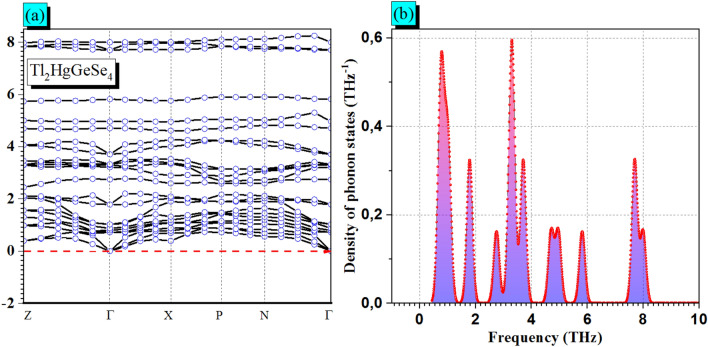
(**a**) The computed phonon band dispersion calculated along several high-symmetry points: $Z\to\Gamma \to X\to P\to N\to\Gamma$ and (**b**) The associated DOS of phonon for tetragonal Tl_2_HgGeSe_4_ crystal.

It is essential to calculate the phonon spectra to comprehend the vibration features of such a compound. So, to check the dynamical stability of the Tl_2_HgGeSe_4_ crystal, we present in Fig. 1a the calculated phonon dispersion along several high-symmetry points: $Z\to\Gamma \to X\to P\to N\to\Gamma$. By analyzing the phonons band spectra it should be noted that all the displayed dispersion curves involve branches with positive frequencies. Data presented in Fig. 1b confirms this suggestion. We demonstrate in Fig. 1b total phonon DOS of atoms constituting the Tl_2_HgGeSe_4_ crystal versus vibration frequencies. There are ten well distinguished peaks. The highest one with phonon DOS intensity of about 0.6 THz^–1^ is centered in vibration frequency of 3.30 THz. Besides, all the pronounced peaks are located at positive frequency range from 0.36 to 8.34 THz, confirming the dynamical stability of the titled crystal.

### Calculated structural parameters

**Figure 2:**
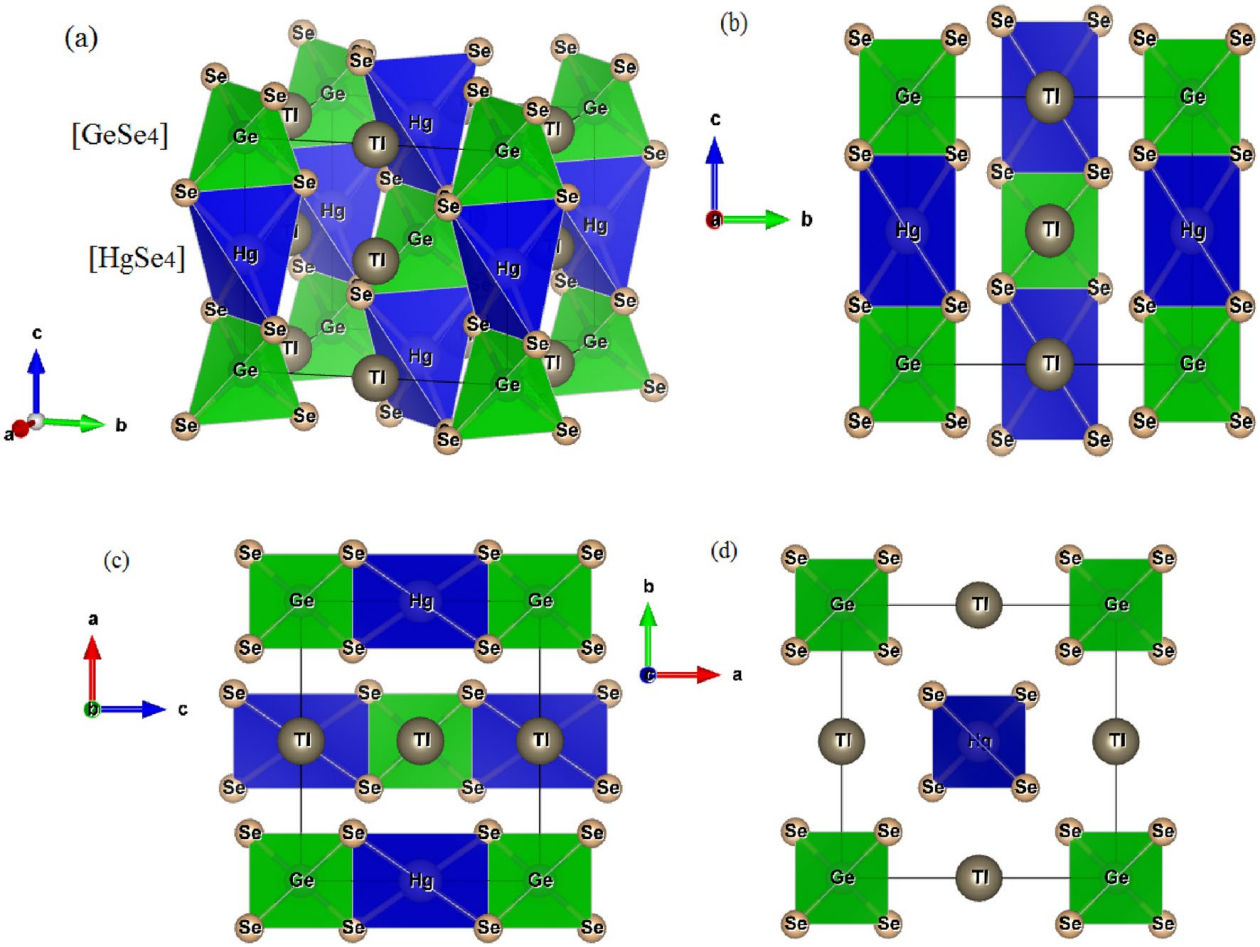
(**a**) The side view of tetragonal Tl_2_HgGeSe_4_ conventional cell, composed with alternative blocks of [HgSe_4_] and [GeSe_4_] tetrahedra with well (Hg and Ge) atom centers remarked. (**b**), (**c**) and (**d**) the atomic arrangement in (cb), (ac) and (ab) planes, respectively.

Based on the XRPD (X-Ray Powder Diffractometer) spectra analysis^47^, one could prove that the quaternary Tl_2_HgGeSe_4_ compound crystallizes in a tetragonal structure, within space group I-42 m (No. 121 in the International X-ray Tables). The conventional cell crystalline structure of the Tl_2_HgGeSe_4_ compound is depicted in Fig. 2a. The blocks of [HgSe_4_] and [GeSe_4_] tetrahedra are obviously stacked alternatively along (cb) and (ac) planes; see Fig. 2b and c. Furthermore, Fig. 2b–d displays different linear and planer atomic densities, in turn help us to better understanding the next elastic and bonding properties.

### Pressure-dependent lattice parameters and elastic constants

#### Lattice parameters and elastic constants at zero pressure

The optimized structural parameters at ambient (zero) pressure of 1 atm ($\sim 0$ GPa), including (a_0_ = b_0_, c_0_), the equilibrium lattice constants, (V_0_) cell volume and (${\rho }_{0}$) mass density were calculated both without and with spin–orbit coupling effect (NSOC and SOC, respectively) and compared to the other calculation and available experimental data. The results are gathered in Table 2.

**Table 2:** The computed equilibrium lattice parameters (a = b and c, in Å), conventional cell volume (*V*_*Cell*_, in Å^3^), the relative deviation of the computed values from the corresponding experimental data related to single-phase crystal (*in* %) in comparative presentation, NSOC(SOC) without(with) including spin–orbit coupling (SOC) via local density approximation (Ceperley and Alder- Perdew and Zunger) LDA(CA-PZ) and generalized gradient approximation of Perdew-Burke-Ernzerhof for solids GGA-PBEsol08.

Functional			a = b	c	V_Cell_	$\rho$
Present work	LDA (CA-PZ)	SOC	7.7438 (− 3.18%)	6.5823 (− 2.69%)	394.723	8.390
		NSOC	7.7455 (− 3.16%)	6.5832 (− 2.64%)	394.949	8.395
	GGA PBEsol08	SOC	7.9057 (− 1.20%)	6.7729 (+ 0.14%)	423.254	7.847
		NSOC	7.9022 (− 1.20%)	6.7564 (− 0.12%)	422.278	7.829
Other^1^			8.0745 (+ 0.95%)	6.9182 (+ 2.72%)		
Experiment		Alloy^2^	7.9947 (− 0.05%)	6.7617 (− 0.04%)	432.170	7.66
		Single crystal^1^	7.9984^**2**^	6.7645^**2**^		

^1^From Vu et al.^1^.

^2^From Mozolyuk et al.^41^.

**Figure 3:**
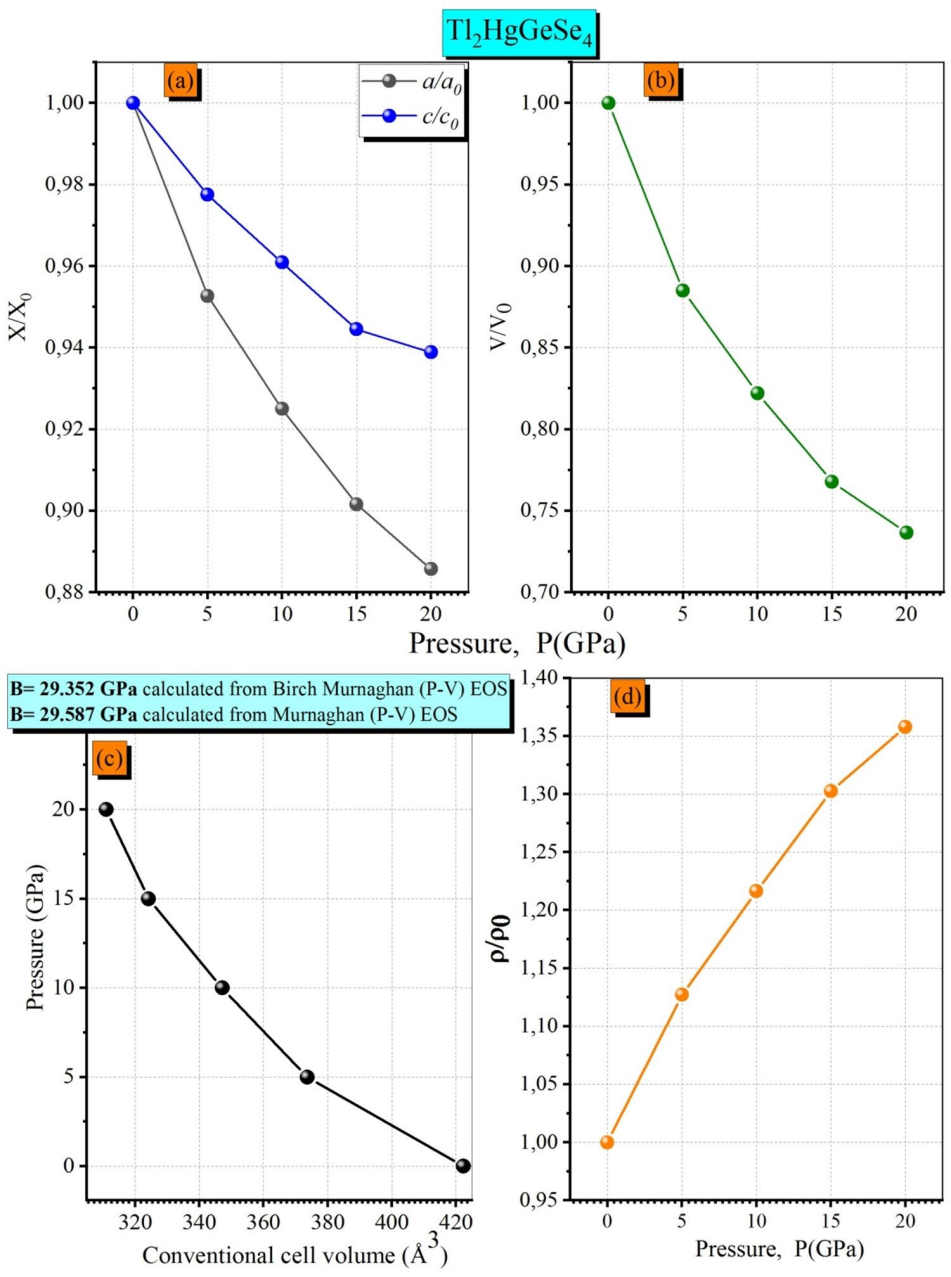
The calculated isopressure-dependence of the normalized lattice parameters (**a**) (a/a_0_ and c/c_0_); (**b**) normalized unit-cell volume V/V_0_, (**c**) conventional cell volume and (**d**) normalized mass density $\rho$/${\rho }_{0}$ for Tl_2_HgGeSe_4_ crystal.

According to data listed in Table 2, the present calculation shows that the deviations of the calculated optimized lattice parameters values (a_0_ = b_0_ and c_0_) from the measured ones (VIZ. single-phase crystal) using GGA-PBEsol08 are about − 1.20% (− 1.20%), and − 0.12% (+ 0.14%) without(with) SOC effect, respectively. With such a relative consistency with experiments, the PP-PW method produces a kind of reliability to predict the optimized atomic coordination. The LDA (CA-PZ) functional is well known for its underestimating the lattice parameters where the deviation in the present work exceeds this value by − 2.64 to − 3.18%. The optimized lattice parameter a is slightly smaller by − 1.20% (− 1.20%) than that derived in Vu and coworker calculation^1^ (+ 0.95%), conversely for the optimized c parameter in which our calculation shows the closest results with respect to the experiment by a deviation of − 0.12% (+ 0.14%). Furthermore, the lattice parameter a_0_(c_0_) value decreases slightly by − 0.05% (− 0.04%) as we go from single crystals to the alloy phase. We illustrate in Fig. 3, the relative changes X/X_0_ of the lattice parameters (a/a_0_, c/c_0_ and V/V_0_) and $\rho$/$\rho$_0_ versus the external applied pressure in a range till to [0–20 GPa] with a step of 5 GPa. The obtained results for a/a_0_, c/c_0_ and V/V_0_ are well fitted to third-order polynomials following the relation:

$$ {X \mathord{\left/ {\vphantom {X {X_{0} }}} \right. \kern-0pt} {X_{0} }} = 1 + \beta xP + \sum\limits_{n = 2}^{n = 3} {K_{n} P^{n} } $$

From Fig. 3a one could remark obviously that when we increase the applied pressure from 0 to 20 GPa with a step of 5 GPa, the ratio c/c_0_ decreases more slowly than a/a_0_. This trend indicates that the considered crystal material is much more compressible along the a-axis than along the c-axis. Further important physical parameters could explain this behavior such as the atomic linear and planar densities shown in Fig. 2b–d where the formers were higher in the (bc) and (ac) planes than at the (ab) one. So, the physical meaning is that in Tl_2_HgGeSe_4_ crystal it is stiffer for strains along the c-axis than along the a-axis. This behavior is attributed to the relative difference in interlayer bond strength along the c-axis compared to that along the a-axis, is proportional to the bonding anisotropy in our system. The computed conventional cell volumes at fixed values of applied hydrostatic pressure are also illustrated in Fig. 3c and fitted to the third-order Murnaghan and Birch-Murnaghan equations of state (P–V EOS). The computed Bulk modulus of the Tl_2_HgGeSe_4_ crystal is about 29.352 GPa and 29.587 GPa when using Birch-Murnaghan and Murnaghan equations of states, respectively (see Fig. 3c). Figure 3d shows also that the normalized mass density $\rho$/${\rho }_{0}$ increases with increasing applied pressure.

#### Elastic features

##### Single-crystal elastic constants and related properties

The knowledge on the elastic constants for such compounds provides an interesting link between the dynamic and mechanic behaviors. However, it gives considerable information concerning the nature of the forces operating in the system. Mainly, for the tetragonal crystal phase, there are six independent stiffness constants Cij(s), namely, C_11_, C_12_, C_13,_ C_33_, C_44_ and C_66_. Until now, no theoretical or experimental values for these quantities are reported in the scientific literature which could help us to construct a comparative estimation. The calculated independent elastic constants Cij(s) of the Tl_2_HgGeSe_4_ crystal are listed in Table 3. Following the values listed in Table 3, the obtained results allow us concluding some interest points.

**Table 3:** The computed elastic constants (*C* ij, in GPa) for the Tl_2_HgGeSe_4_ crystal without(NSOC)/with(SOC) the inclusion of the spin–orbit coupling effect.

	Elastic constant Cij					
	C_11_ = C_22_	C_12_	C_13_	C_33_	C_44_	C_66_
NSOC GGA PBE-sol08	42.91	21.06	18.66	72.92	11.27	10.33
SOC GGA PBE-sol08	43.17	21.18	19.03	72.71	13.20	13.96

First of all, we try to check the Born-Huang stability criteria while in the tetragonal phase it should obey four conditions^48^:

*C*_11_ >*|C*_12_*|*; 2(*C*_13_)^2^ < *C*_33_ (*C*_11_ + *C*_12_), *C*_44_ > 0 and *C*_66_ > 0.

The predictive elastic constants C_ij_(s) satisfy all the above-mentioned conditions which indicate the mechanical stability of the Tl_2_HgGeSe_4_ crystal. Furthermore, the elastic constants C_11_ and C_33_ traduce the materiel stiffness toward uniaxial stress along the crystallographic (a or b) and c axes, respectively. In our case, the C_11_ constant is smaller than C_33_; this means that the crystal under investigation is less resistant to applied stress along the [100] and [010] crystal directions than along the [001] direction. This fact is in good agreement with the previous findings presented in Fig. 2b–d. The knowledge on the elastic sound velocity in such medium is essential for different scientific and engineering applications and serves to give more details about material compactness, elastic modulus, thermal conductivity, homogeneity and so on. The acoustic wave velocities along different directions in a crystal can be obtained by solving the Christoffel equation^49^. The calculated sound wave velocities are tabulated in Table 4.

**Table 4:** The calculated SOC-(GGA-PBEsol08) independent single-crystal elastic sound wave velocities for some different propagating crystallographic directions (V, in m/s) for Tl_2_HgGeSe_4_ crystal.

Direction	[100]			[110]			[001]					
Velocity	v_L_ [100]	V_T1_ [010]	V_T2_ [001]	v_L_ [110]	V_T1_ [0–10]	V_T2_ [001]	v_L_ [001]	v_T_ (100)	*V*_*l*_	*V*_*t*_	*V*_*m*_	${\theta }_{D}$
SOC-GGA PBE-sol08	2346	1334	1297	2425	1184	1297	3044	1297	2506	1350	1494	150 K

The subscripts L and T stand for the longitudinal and transversal polarization of the sound wave, respectively. The mass density *ρ* is equal to 7.847 g/cm^3^. The velocity expression in each case can be found in Ref.^25^.

From Table 4, one can realize that the estimated transversal sound wave propagates with a velocity less than the corresponding longitudinal one, whatever its polarity, resulting to indicate an anisotropy in velocity propagation.

Furthermore, the crystals of the Tl2HgGeSe4 compound with a higher velocity along the longitudinal c direction [001] (V_L_[001] = 3044 m.s^–1^) confirming the hardness of the crystal along the c-axis as we have already discussed vide supra.

**Figure 4:**
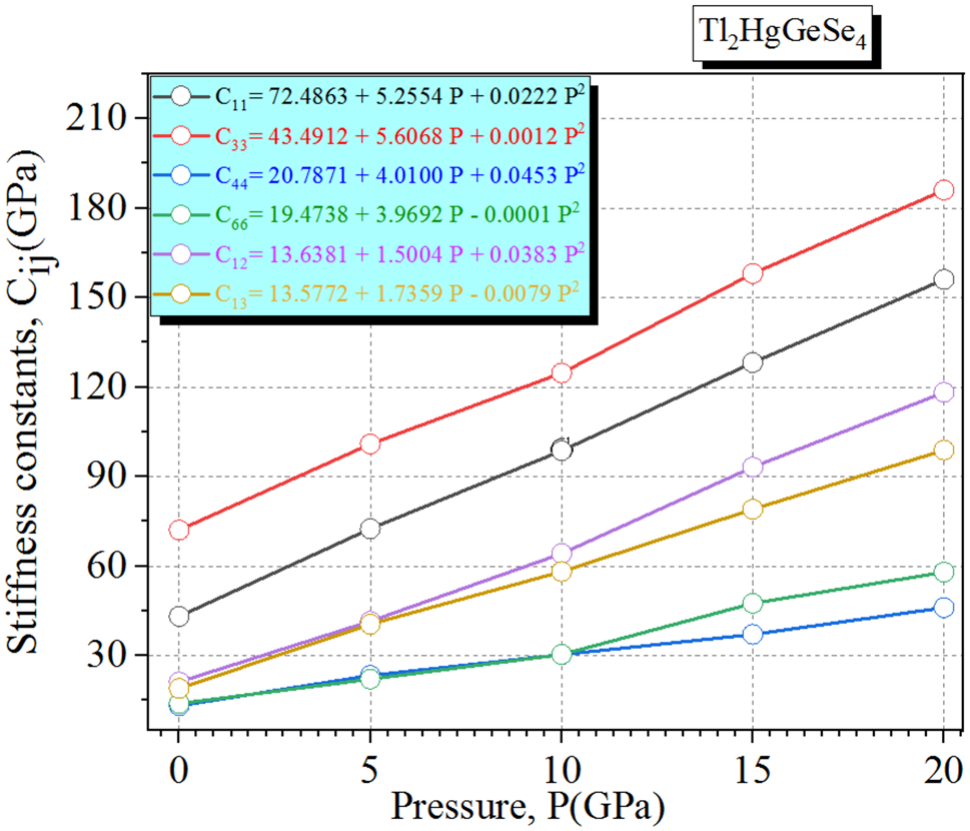
Pressure-dependent elastic constants Cij_(S)_ for the I-42 m Tl_2_HgGeSe_4_ crystal, where we show symbols which refer to the computed Cij(s) results within SOC GGA-PBE sol08 functional and the continuous lines stand for the second-order polynomial fits ($C_{ij} (P) = a_{1} + a_{2} P + a_{3} P^{2}$).

Let’s delve into more details about the elastic properties of Tl_2_HgGeSe_4_. The pressure dependent-elastic constants C_ij_ are illustrated in Fig. 4.

Figure 4 discloses that all the main six elastic constants of the tetragonal phase (C_11_, C_12_, C_13_, C_33,_ C_44_ and C_66_) increase monotonously with the increasing pressure. The elastic constants C_11_ and C_33_ physically traduce the elasticity in length unit in which they change with the longitudinal strain. The elastic constants C_12_, C_13_ and C_44_ are proportional to the shape elasticity. We can see a relatively slight difference in behaviors associated with the considered elastic constants. Also, one can judge that the mechanical stability under external iso-pressure via seven conditions is following:

*C*_11_ > *C*_12_…(a), *2C*_13_ – *C*_33_ < *C*_11_…(b), C_44_ > 0…(c), *C*_66_ > 0…(d),

*C*_33_ > 0…(e), *C*_11_ > 0…(f), *2C*_11_ + *2C*_12_ + *C*_33_ > 2C_13_…(g).

**Figure 5:**
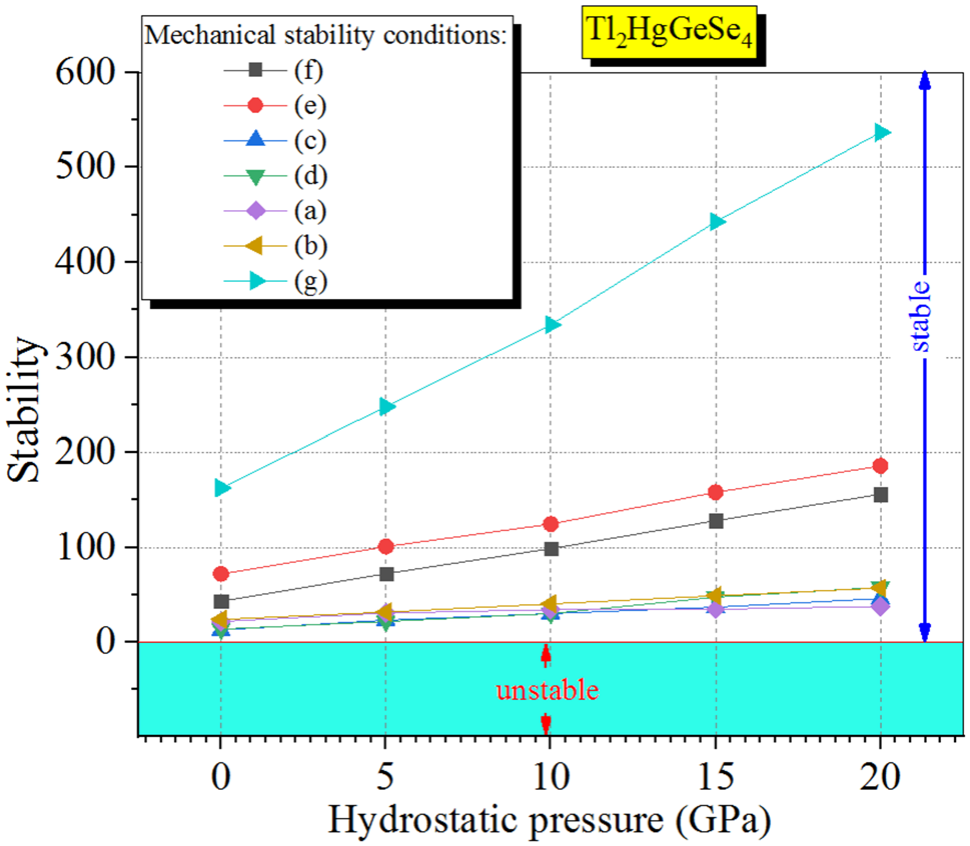
The pressure dependence mechanical stability conditions of the I-42 m Tl_2_HgGeSe_4_ crystal at 0 K temperature.

The obtained results related to the mechanical stability studies for the entitled compound following the above expressions are plotted in Fig. 5. One can see obviously the mechanical stability of the system under study along all [0 to 20] GPa pressure range. These results strongly support the predictive phonon spectra interpretation.

##### Elastic anisotropy and microcracks

In the present work, we aim also to check the anisotropy of the material. The visualization of three-dimensional (3D) stereograms for Young modulus and compressibility regarded as a practical method to probe the anisotropy of a system along its principle crystal directions. In such a 3D representation, any isotropic system would exhibit a spherical shape. Therefore, other different cases indicate the elastic anisotropy. Young’s modulus (E) and compressibility (${\varvec{\beta}}$) expressions in three dimensional closed surfaces are given by the following equations: $E \, = \, \frac{1}{{\left( { \, l_{1}^{4} S_{11} + l_{2}^{4} S_{22} + l_{3}^{4} S_{33} + 2l_{1}^{2} l_{2}^{2} S_{12} + 2l_{1}^{2} l_{3}^{2} S_{13} + 2l_{2}^{2} l_{3}^{2} S_{13} + (l_{2}^{2} l_{3}^{2} + l_{1}^{2} l_{3}^{2} )S_{44} \, + \, l_{1}^{2} l_{2}^{2} S_{66} } \right)}}$

$$ \beta \, = { (}S_{11} + S_{12} + S_{13} ) - { (}S_{11} + S_{12} - S_{13} - S_{33} )l_{3}^{2} $$

where $S_{ij}$ stands for the calculated compliance constants, while $l_{1}$, $l_{2}$ and $l_{3}$ are the directional cosines respecting the x, y and z principle axes, respectively.

**Figure 6:**
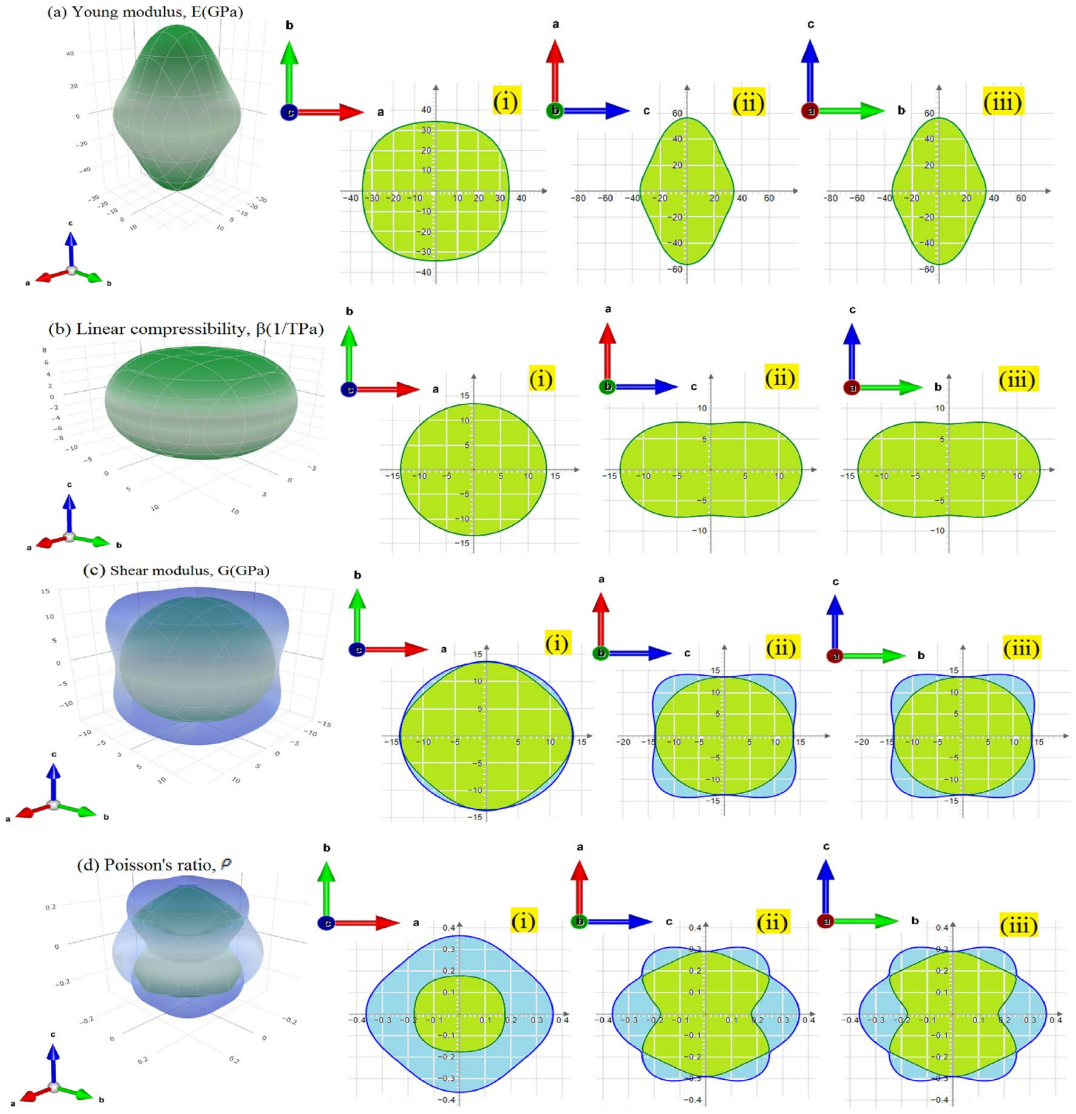
(**a**–**d**) the calculated 3D stereogram of Young’s modulus (E in GPa), the compressibility ($\beta$ in TPa^-1^), Shear modulus (G in GPa) and Poisson’s ratio (dimensionless quantity), respectively. Each mechanical parameter shown alongside with it’s cross-sectional in different planes (**i**), (**ii**) and (**iii**) which stand for (**ab**), (**ac**) and (**cb),** respectively, for the quaternary Tl_2_HgGeSe_4_ compound. The disclosed blue/green (3D surfaces) and (2D solid lines) refer to maximum/minimum magnitudes of the reported mechanical parameters, respectively.

In Fig. 6, we show the (3D) spatial representation of the crystal principle direction-dependent Young modulus and compressibility with their projection (cross-sectional) of Tl_2_HgGeSe_4_ crystal in different planes. The shapes of spatial young modulus (E), linear compressibility($\beta$), shear modulus (G) and poisson’s ratio ($\rho$) diverge from the spherical one. Further, young modulus (E) and compressibility are characterized by themes maximum and minimum values ($E^{max}$ and $E^{\min }$) and ($\beta^{max}$ and $\beta^{\min }$) being equal to (62.032 and 28.812)GPa and (13.672 and 6.7175)TPa^-1^, respectively, indicating a visible elastic anisotropy in our case (See Fig. 6a and b).

Owing to the relative mechanical anisotropy in both Young modulus and compressibility, the micro cracks MC_(s)_ as a type of material failure are widely induced at the upper layers of the crystal under consideration. These MC_(s)_ act to alter the atomic arrangement and consequently random crystal orientation may appear at the surface which could be the reason for the multicolor remarkable (dark blue, green, light pink and violet).

Furthermore, following the previous illustrations of the four mechanical modulus (See Fig. 6), one can efficiently express the limits of these quantities and also compare the anisotropy remarked statistically as shown in Table 5.

**Table 5:** The variations (minimum and maximum) values of Young’s modulus (in GPa), compressibility (in TPa^−1^), shear modulus (in GPa), Poisson’s ratio (dimensionless quantity) and theirs anisotropy magnitude for tetragonal Tl_2_HgGeSe_4_ compound.

	Young’s modulus (E)		Linear compressibility (β)		Shear modulus(G)		Poisson’s ratio ($\rho$)	
	*E*_min_	*E*_max_	β_min_	β_max_	*G*_min_	*G*_max_	$\rho$_min_	$\rho$_max_
Value	28.812	62.032	6.7175	13.672	10.328	17.363	0.1408	0.44979
Anisotropy (A)	2.153		2.0352		1.681		3.1946	

Further, with the aid of ELATE (elastic tensor analysis), one could recount certain quantitative studies in which reporting the minimum and maximum values of the previous elastic modulii. The results gathered in Table 5. The anisotropy (A) calculation of the elastic moduli also is implied by the following ratio: A = X_max_/X_min_. The highest value of the anisotropy (A) to the lowest one takes the order of: ${\varvec{\rho}}\to {\varvec{E}}\to$ **β **$\to {\varvec{G}}$**.**

### Electronic properties

#### Energy band dispersions

**Figure 7:**
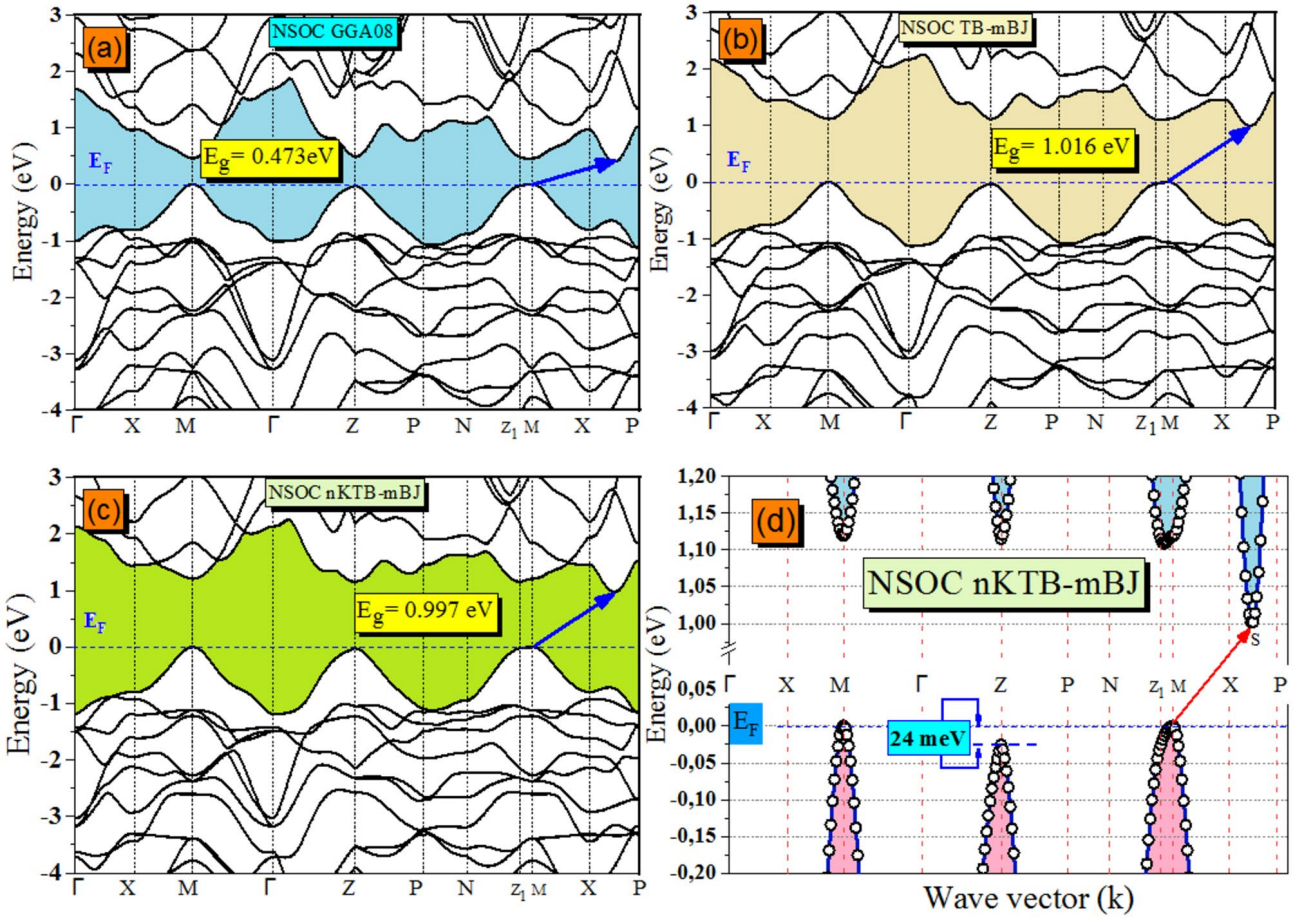
The computed (**a**) GGA08, (**b**) TB and (**c**) new Koller, Tran and Blaha modified Becke and Johnson (nKTB-mBJ) electronic band structure for Tl_2_HgGeSe_4_ without spin–orbit coupling effect. (**d**) The extended form of (**c**) as a prototype.

**Figure 8:**
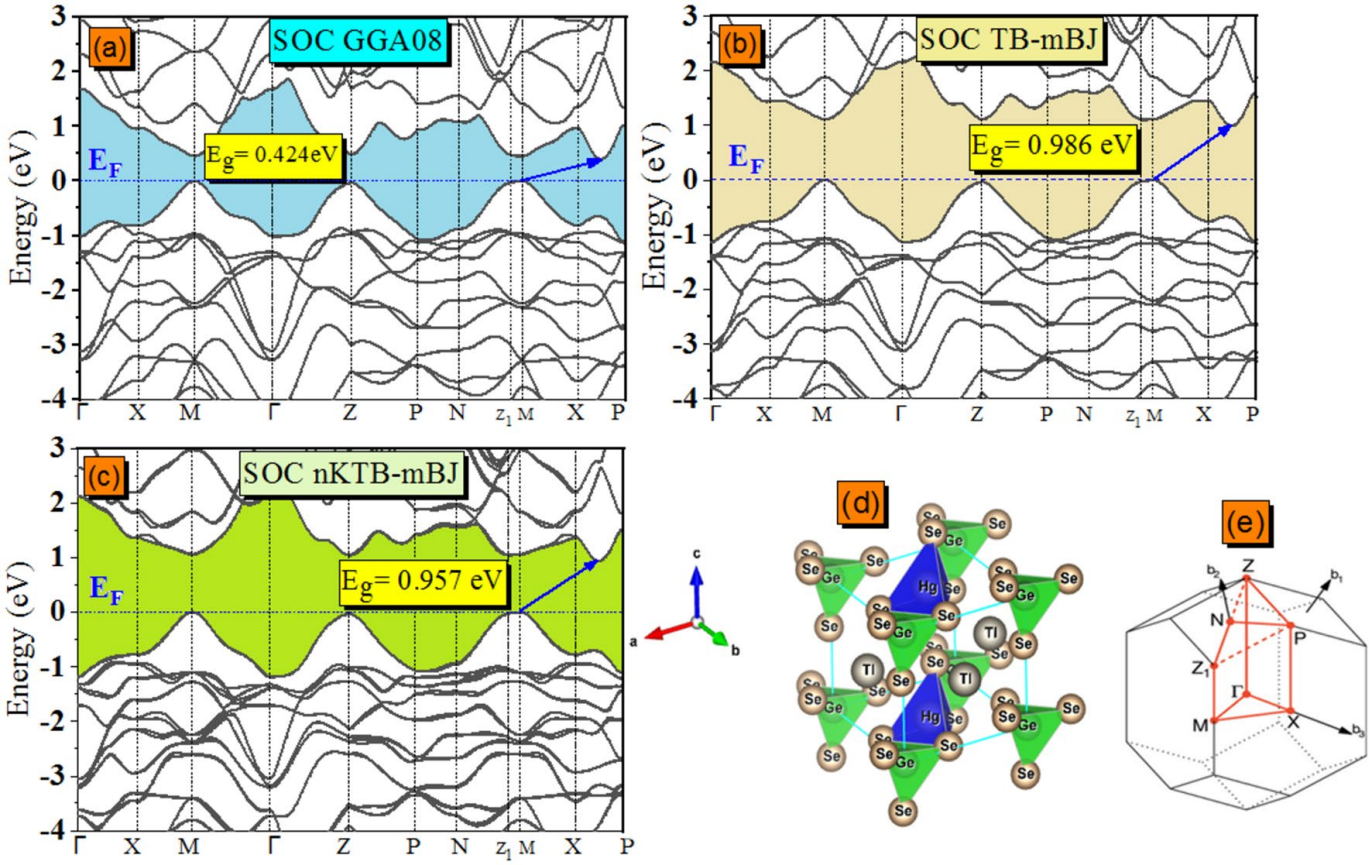
The computed (**a**) GGA08, (**b**)TB and (**c**) new KTB-mBJ electronic band structure for Tl_2_HgGeSe_4_ including the spin–orbit coupling effect. (**d**) The side view of Tl_2_CdGeSe_4_ atomic arrangement of the primitive cell. (**e**) The associated first Brillouin zone of BCT_1_(body-centred tetragonal “1” where ${\text{c}}\langle {\text{a}}$). The high-symmetry K-points for our computed band structures are set as the following: ${\Gamma }\left( {0.00 \,\,0.00 \,\,0.00} \right) \to {\text{X}}\left( {0.00\,\, 0.00 \,\,0.50} \right) \to {\text{M}}\left( { - 0.50 \,\,0.50 \,\,0.50} \right) \to {\Gamma }\left( {0.00\, \,0.00 \,\,0.00} \right) \to {\text{Z}}\left( {0.43 \,\,0.43 \,\,{-}0.43} \right) \to {\text{P}}\left( {0.25\,\, 0.25\,\, 0.25} \right) \to {\text{N}}\left( {0.00 \,\,0.50 \,\,0.00} \right) \to {\text{Z}}_{1} \left( { - 0.43\,\, 0.57\,\, 0.43} \right) \to {\text{M}}\left( { - 0.50 \,\,0.50\,\, 0.50} \right) \to {\text{X}}\left( {0.00 \,\,0.00\,\, 0.50} \right) \to {\text{P}}\left( {0.25\,\, 0.25 \,\,0.25} \right)$.

According to the electronic band structure calculated by Vu and coworkers^1^ using various potentials (GGA-PBE, GGA-PBE + U, TB-mBJ and TB-mBJ + U + SO) and in order to verify these results, we repeat this calculation starting on our previously announced geometric optimizations along the lines of high symmetry points in the first Brillouin zone (BZ) in the $X\to M\to\Gamma \to Z\to \text{N}\to P\to X$ sequence. The obtained band structures (not shown here) reveal their similarly to those presented in Ref.^1^ but we draw the reader’s attention to interesting points of view: the valence band maximum (VBM^ax^) is still more dispersive than the conduction band minimum(CBM^in^). The CBM^in^ is detected at the intermediate high symmetry points positioning between X and P (for clarity, we refer to the S point here). The present data indicate that the VBM^ax^ possesses two wide tops at almost equivalent energy levels. In Ref.^1^. It is indicated that the VB topmost point occurs at Z point, but in fact, both the present graphical values and the extended band gaps previously displayed by Vu et al. show that the topmost may occur at M point with a difference reaching about tenths of meV comparing to the Z point top. So, to explain these differences, we can refer to the article by Setyawan et al.^49^ which suggests that, depending on the tetragonal lattice parameters (a > c) and (c > a), the primitive cell has two possible shapes (i.e. consequently two different coordinates of the symmetry points resulting to construct two different paths) of the BZ denoted BCT_1_ (Body Centred Tetragonal 1, Zn_2_(SiO_4_) band structure as a prototype) and BCT_2_ (Body Centred Tetragonal 2, CaIn_2_O_4_ band structure as a prototype), respectively. In our case, the BCT_1_ was chosen and the lines of high symmetry points in (BZ) are as follow: $\Gamma \to \text{X}\to \text{M}\to\Gamma \to \text{Z}\to \text{P}\to \text{N}\to {\text{Z}}_{1}\to \text{M}\to \text{X}\to \text{P}$. The calculated band structures using (GGA-PBEsol08, TB-mBJ and new KTB-mBJ (nKTB-mBJ)) models without the SOC effect are depicted in Fig. 7a, b and c and with SOC in Fig. 8a–c. The difference in energy level of the two near tops in the VBM^ax^ shown in Fig. 7d is equal to 24 meV using NSOC new KTB-mBJ functional as a prototype. The results show the forbidden energy band with an indirect type (M $\to$ S) for any exchange correlation potential used without or with the SOC effect. Figure 7d and e illustrates the tetragonal primitive cell and their associated (BZ) shape with (c < a), respectively.

**Figure 9:**
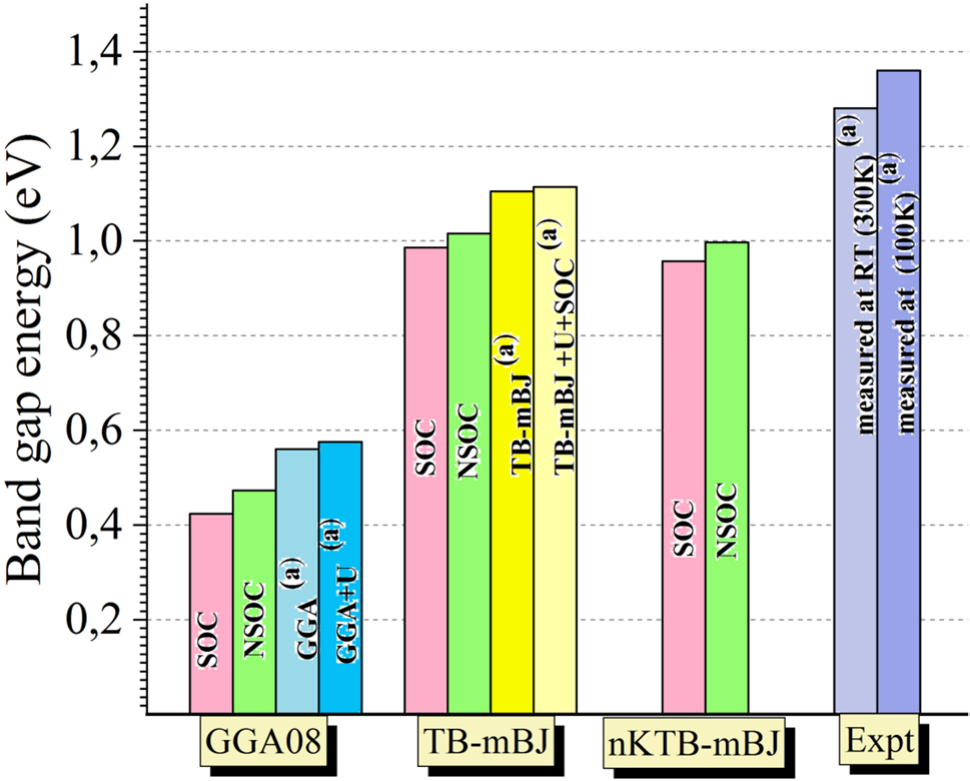
Comparative illustration of the Tl_2_HgGeSe_4_ optical band gap calculation using NSOC/SOC (GGA-PBE08, TB-mBJ and new KTB-mBJ) functionals. ^a^Theoretical Values and experimental ones from Vu et al.^1^ (E.g. in eV).

In order to examine the effect of the inclusion of various exchange correlation potentials at the obtained electronic properties such as the electronic band dispersion, we present in Fig. 9 the results of a statistical study of energy band gap values without and with the SOC effect, for comparison. From Fig. 9, one can notice that all the present theoretical results relatively underestimate the forbidden energy band in this sequence GGA-PBEsol08 $\to$ new KTB-mBJ $\to$ TB-mBJ by -63.05%, -22.11%, and -20.63% without the SOC effect and by − 66.88%, − 25.23% and − 22.97% with SOC effect compared to the RT experimental value (E_g_ = 1.28 eV at 300 K). Soni et al.^50^ and Mezelit et al.^51^ report that the use of new KTB-mBJ approach tends to minimize more the underestimations which have been observed in TB-mBJ and GGA08 functionals for transition metal-based halides (Cs_2_ZSbX_6_) double pervoskite and Y_x_Al_1−x_N semiconductor alloys, respectively. From Fig. 9, the predicted band gaps employing the modified Tran-Blaha potential show the closest values to the RT experiment with the smallest difference (− 20.63% via NSOC effect), followed by calculations with new KTB-mBJ and then with the GGA-PBEsol08 functional.

Additionally, the experimental forbidden band measurements indicate that E_g_ tends to vary with + 80 meV when the system temperature decreases from RT to 100 K obeying the Varshni empirical relationship^52^.

#### Charge carrier effective masses

The effective charge-carrier mass (electrons and holes) is a fundamental quantity used in a great number of experimental analyses and as a crucial key in theoretical models of semiconductors. The electron effective mass is among the main factors that describe the magnitude of electrical conductivity and efficiency in transport properties for such a system. Based on the calculated electronic band structures, the effective (electrons and holes) masses are evaluated. We employed such a fitting at the E = f($\overrightarrow{k}$) around the extremes of intended parabolas. The charge effective mass ($m^{*}$) is calculated via the following expression:

$$ \frac{1}{{m_{e,h}^{{^{*} }} }} = \left[ {\frac{{4\pi^{2} }}{{{\text{h}}^{{2}} }}\frac{{\partial^{2} E(k)}}{{\partial^{2} k}}} \right]m_{0} $$

where $m_{e,h}^{{^{*} }}$, $m_{0}$ and h are the (electron and hole) effective mass, electron rest mass and Plank constant, respectively. It is worth indicating that the electron effective masses for Tl_2_HgGeSe_4_ compound are calculated at the bottom energy level of the conduction band while the hole effective masses are calculated at the top energy level of the valence band. All the results are gathered and tabulated in Table 5.

According to data listed in Table 6, the charge effective mass values ($m_{e}^{{^{*} }}$ and $m_{h}^{{^{*} }}$ ) vary considerably when the direction in high symmetry changes showing a relative anisotropy in charge-carriers mobility. Besides, the computed holes effective masses are lighter than those of electrons indicating the p-type electroconductivity of the Tl_2_HgGeSe_4_ crystal confirming the experimental findings.

**Table 6:** The computed electron ($m_{e}^{{^{*} }}$) and hole ($m_{h}^{{^{*} }}$) effective masses for Tl_2_CdGeSe_4_, at the top of the valence band and at the bottom of the conduction band, along the (S $\to$ P and S $\to$ X) and (M $\to$ X and M $\to$ Z_1_) directions in the BZ, respectively, considering the spin orbit coupling effect.

Exchange–correlation potential	Electron effective masses ($m_{e}^{{^{*} }}$)		Hole effective masses ($m_{h}^{{^{*} }}$)	
	S $\to$ P	S $\to$ X	M $\to$ X	M $\to$ Z_1_
GGA-PBEsol08	0.456	0.424	0.342	0.335
TB-mBJ-GGA	0.718	0.712	0.413	0.385
New Koller TB-mBJ-GGA	0.543	0.522	0.379	0.366

The values are given in the unit of free electron mass m_0_.

**Figure 10:**
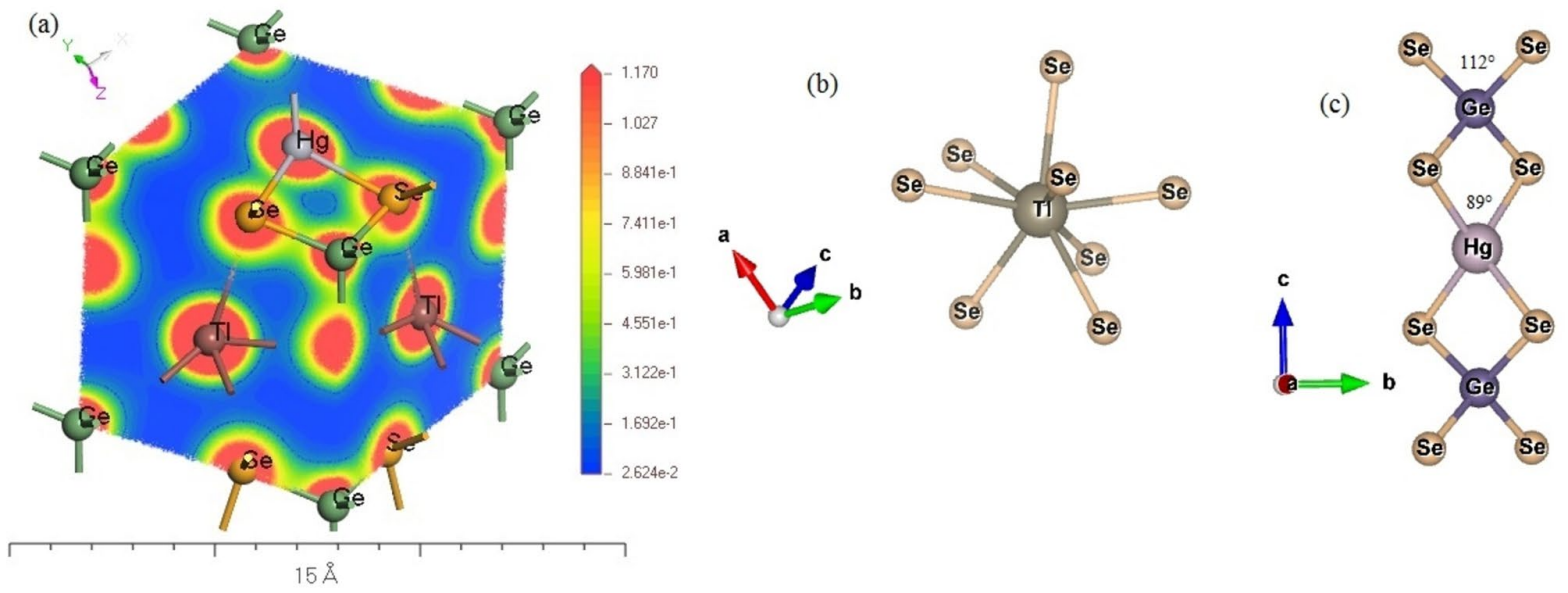
(**a**) The charge contour plots for Tl_2_HgGeSe_4_ primitive cell. High electron density colored in red regions and it is low in the blue regions. (**b**) Eight selenium atoms are bonded to Thallium atom in quaternary Tl_2_HgGeSe_4_ compound. (**c**) The adjacent surrounding atoms to Germanium (Ge) and mercury (Hg). The angels formed between bonds (in degree).

The chemical bond property of the considered compound was probed via the charge density distribution map in the Tl_2_HgGeSe_4_ primitive cell, as shown in Fig. 10a. From Fig. 10b it apparent that, each Tl atom has got eight bonds with selenium atoms (Tl-Se). Figure 10a also indicates that the Tl-Se bonds are ionic (i.e. the spherical charge distribution around thallium atoms reveal an ionic bond nature) with respect to the total ionic radii (presented in Table 7) d_*R*(Tl_^+^_)+*R*(Se_^2–^_)_ = 3.4 Å. This result also fairly agrees with our SOC calculations (3.2822–3.5090) Å, others theoretical (3.3528–3.5848) Å and experiment (3.3230–3.5230) Å data reported by Mozolyuk et al. ^21^. Also, Fig. 10c shows that the intralayer bonding in [HgSe_4_] and [GeSe_4_] tetrahedra are composed by Hg-Se and Ge-Se bonds, respectively. The total ionic radii (d_*R*(Hg_^2+^_)+*R*(Se_^2–^_)_ and d_*R*(Ge_^4+^_)+*R*(Se_^2–^_)_) are larger than the experimental ones indicating the predominant covalent character for both Hg-Se and Ge-Se bonds. In general, the chemical bonds in the Tl_2_HgGeSe_4_ crystal are mixture of covalent and ionic bonds. The [HgSe_4_] tetrahedra in Fig. 10c (see also Fig. 2a–c) are thicker than [GeSe_4_], which could be explained by the chemical architecture of Tl_2_HgGeSe_4_ tetragonal crystal, where the longer bonds Hg-Se in the tetrahedral [HgSe_4_] form angles (Se-$\widehat{\text{Hg}}$-Se) of about 89°, conversely to the shorter bonds Ge-Se in [GeSe_4_] slabs which form angels (Se-$\widehat{\text{Ge}}$-Se) with nearly 112°. Furthermore, the electronegativity difference (ED) is a helpful factor to measure the stiffness of the bonds under study^53,54^. The highest (ED) value has the weak bond, i.e. ${\Delta \chi }_{Ge-Se}=0.54<{\Delta \chi }_{Hg-Se}=0.55<{\Delta \chi }_{Tl-Se}=0.93$. From the latter ED estimations, the strongest bonds in the title compound are observed between Ge and Se species (Ge-Se bonds) and that leads us to conclude that the [GeSe_4_] are more hardened compared to [HgSe_4_] ones.

**Table 7:** Electronegativity of the chemical elements constituting the Tl_2_HgGeSe_4_ crystal, in Pauling scale (dimensionless), R(X) represents the ionic radius (in Angstrom).

Element	Tl		Hg	Ge	Se
Electronegativity ($\chi$)	1.62		2.00	2.01	2.55
Ionic radius (Å)	*R*(Tl^+^) = 1.49		*R*(Hg^2+^) = 1.12	*R*(Ge^4+^) = 2.72	*R*(Se^2–^) = 1.91
Bond length (Å)	Theory			Expt	
	Present work		Other		
	NSOC	SOC			
d_Hg-Se_	2.6718	2.6970	2.7540	2.688 (2)	
d_Ge-Se_	2.3818	2.3945	2.4443	2.420 (2)	
d_Tl-Se_	3.2613 3.4911	3.2822 3.5090	3.3528 3.5848	3.323 (3) 3.523 (2)	

The calculated bond lengths (d _X-X_) evaluated taking into account the SOC effect in comparison with the experimental ones (in Angstrom), the number between brackets stands to the identical bonds (N.B.).

### Optical properties

**Figure 11:**
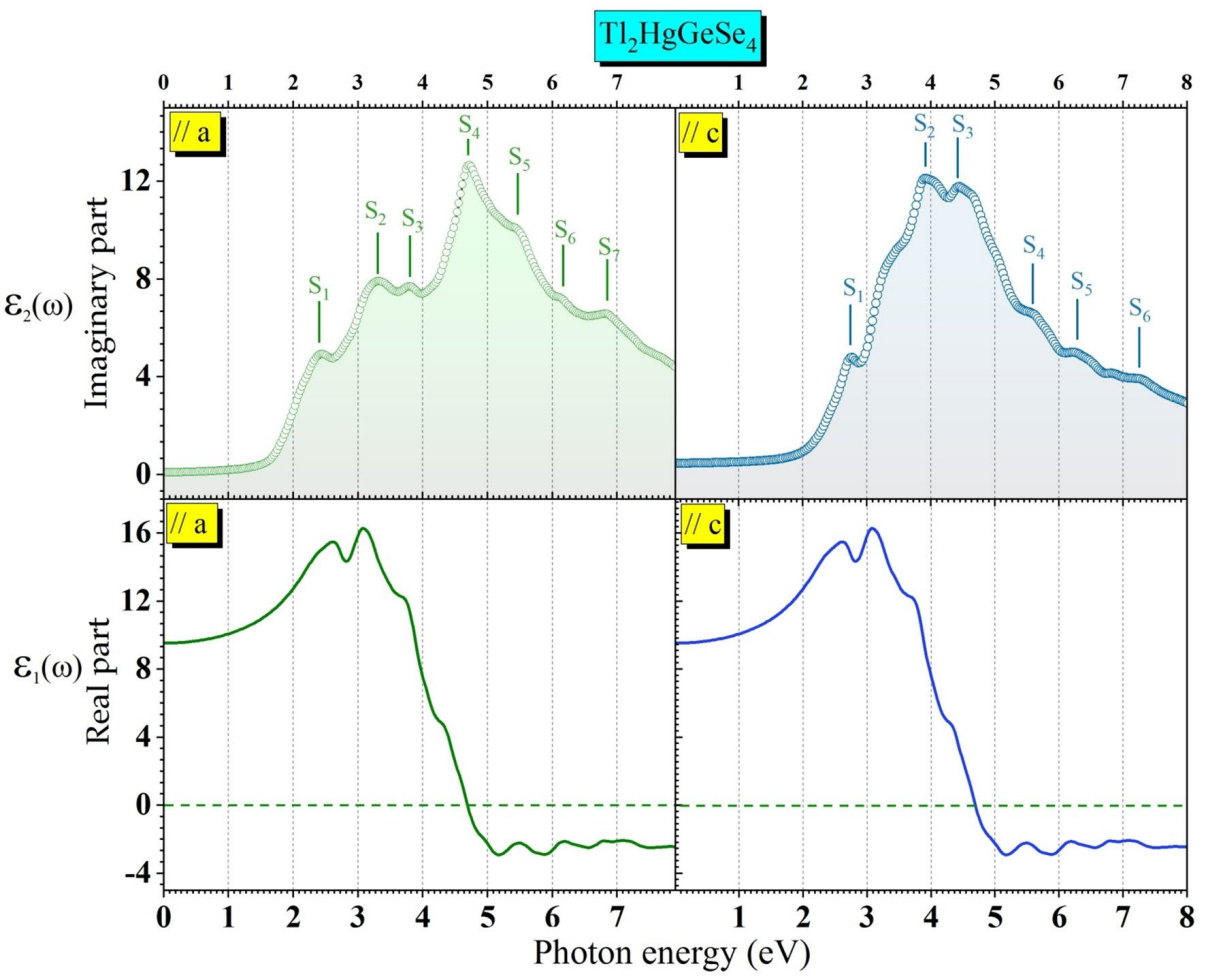
Frequency dependent the imaginary (*ε*_2_(*ω*)) part: up panel, and real (*ε*_1_(*ω*)) part: down panel of the dielectric function versus photonic radiation (E_photon_, in eV), parallel to the *a*- (left panel) and parallel to the* c*-axes (right panel) for the Tl_2_HgGeSe_4_ compound. The structures top label S_*i*_ (*i* = 1, 2, 3…).

It is of great importance to study the optical features of such material when it is synthesized for the first time. This tendency is driven by our desire to comprehend how the system interacts with electromagnetic radiation. There are several optical parameters are of great importance to understanding the optical behavior of our system. Among these important optical parameters is the dielectric function. It is the involvement key which encompasses other linear optical parameters proportional to the behavior of light (VIZ. reflectivity $R(\omega )$, transmittance $T(\omega )$, absorbance $A(\omega )$, refractive index $n(\omega )$…etc.). The dielectric function is given by the following expression:

$$ \varepsilon (\omega ) = \varepsilon_{1} (\omega ) + i\varepsilon_{2} (\omega ) $$

where $\varepsilon_{1} (\omega )$ and $\varepsilon_{2} (\omega )$ are the real part and the imaginary part of the dielectric function $\varepsilon (\omega )$, respectively. The photon dispersion is described by the real part while the photon absorption is described by the imaginary part of the dielectric function. The components ($\varepsilon_{2}^{ij} \left( \omega \right)$ and $\varepsilon_{1}^{ij} \left( \omega \right)$) of imaginary $\varepsilon_{2} (\omega )$ and real $\varepsilon_{1} (\omega )$ parts are given by the following expressions^55–57^:

$$ \left\{ {\begin{array}{*{20}c} {\varepsilon_{2}^{ij} \left( \omega \right) = \frac{{Ve^{2} }}{{2\pi \hbar m_{0}^{2} \omega^{2} }}\sum\limits_{{n,n^{/} }} {\int {d^{3} k\left\langle {k_{n} } \right|P_{i} \left| {k_{{n^{/} }} } \right\rangle \times \left\langle {k_{{n^{/} }} } \right|P_{j} \left| {k_{n} } \right\rangle f_{kn} \left( {1 - f_{{k\omega^{\prime}}} } \right)\delta \left( {E_{{k\omega^{\prime}}} - E_{kn} - \hbar \omega } \right)} } } \\ {\varepsilon_{1}^{ij} \left( \omega \right) = 1 + \frac{2}{\pi }\int\limits_{0}^{\infty } {\frac{{\varepsilon_{2}^{ij} (\omega^{\prime})\omega^{\prime}d\omega^{\prime}}}{{\omega^{*2} - \omega^{2} }} \, } } \\ \end{array} } \right., $$

where $\hbar \omega$ stands for the incident photons energy. $\left| {k_{n} } \right\rangle$ is the wave function of the occupied electronic state ($\overrightarrow{k}\to n$) with energy of $E_{kn}$ in the valence band of index n with a wave vector $k$ $\left| {k_{{n^{/} }} } \right\rangle$ is the wave function of an unoccupied electronic state (${\overrightarrow{k}}^{/}\to {n}^{/}$) corresponding to the energy $E_{{kn^{/} }}$ in the conduction band with a wave vector $k$. ${P}_{\text{i}}$ $(i = x, y, z)$ is the momentum operator, $f_{kn}$ is the Fermi distribution corresponding to the energy level $E_{kn}$ and $\delta {(}E_{{kn^{/} }} - E_{kn} - \hbar \omega {)}$ is the Dirac function. According to the tetragonal symmetry of the Tl_2_HgGeSe_4_ crystal, $\varepsilon_{1} (\omega )$ and $\varepsilon_{2} (\omega )$ have two diagonal components ($\varepsilon_{1}^{//a} (\omega )$ and $\varepsilon_{1}^{//c} (\omega )$) and ($\varepsilon_{2}^{//a} (\omega )$ and $\varepsilon_{2}^{//c} (\omega )$), respectively. Figure 11 depicts spectra of the $\varepsilon_{1} (\omega )$ and $\varepsilon_{2} (\omega )$ components as a function of the incident photon energy [0–8] eV.

From Fig. 11 the calculated spectra are not similar indicating a noticeable anisotropy in the crystal under study. Besides, the microscopic origin of any electronic transition from the occupied states in the valence band (V_i_(k)) to the other empty states in the conduction band (C_j_(k)) is well shown. Following a precise and sophisticated treatment^46,58–61^ at the imaginary components parallel to a ($\parallel a$) and parallel to c ($\parallel c$), the fine structures in $\varepsilon_{2}^{//a} (\omega )$ and $\varepsilon_{2}^{//c} (\omega )$ spectra denoted S_i_ and their localization, individual band-to-band contribution, their position and the location of the corresponding wave vectors (k) in the first Brillouin zone (BZ) are tabulated in Tables 8 and 9. Further clarification, our treatments focused on structure S_1_ related to Fig. 11//a show five well-identified contributions in the formation of the main structure S_1_ (see Table 8), each contribution presents a well identified electronic transition as follows: V_1_-C_1_, V_2_-C_1_, V_3_-C_1_, V_1_-C_2_ and V_1_-C_3_. Detailed information about the main contributors originating the remaining fine-structure peculiarities (S_2_, S_3_, S_4_, S_5_, S_6_ and S_7_) in the 1^st^ Brillouin zone regions are tabulated in Tables 8 and 9.

**Table 8:** Peak locations of the ${\mathcal{E}}_{2}^{\parallel a}(\omega )$ spectrum with the dominant interband transition (IT)_s_ contributions to the considered structure (S_i_).

Structure	Location	Band to band transition	Sub-peak position	Brillouin zone region
S_1_	2.38	V_1_–C_1_	2.74	Z-X-M, N-Z_1_-M, X-P
		V_2_–C_1_	2.56	Z-X-M, N-Z_1_-M, X-P
		V_3_–C_1_	2.51	Z-X-M-Γ-Z, Z_1_-M-X-P
		V_1_–C_2_	2.73	Z-X-M, Γ-Z-P, M-X-P
		V_1_–C_3_	2.82	Z-X-M, Z_1_-M-X-P
S_2_	3.28	V_1_–C_2_	3.80	Z-X-M, Γ-Z-P-N, X-P
		V_2_–C_2_	3.90	Z-X-M, N-Z_1_-M, X-P
		V_3_–C_1_	3.19	Z-X-M, Z_1_-M-X-P
		V_3_–C_2_	3.90	Z-X-M, Z_1_-M-X-P
		V_4_–C_1_	3.47	Z-X-M, N-Z_1_-M-X-P
		V_4_–C_2_	3.96	Z-X-M, Z_1_-M-X-P
S_3_	3.80	V_6_–C_5_	4.62	Z-X-M, N-Z_1_-M-X-P
		V_8_–C_4_	4.27	Z-X-M, N-Z_1_-M-X-P
		V_7_–C_1_	4.50	Z-X-M, Z-P-N, Z_1_-M-X-P
		V_5_–C_2_	4.69	Z-X-M, Γ-Z-P, Z_1_-M-X-P
		V_5_–C_3_	4.72	Z-X-M, Z_1_-M-X-P
S_4_	4.66	V_1_–C_6_	5.10	Z-X-M, P-N-Z_1_-M, X-P
		V_2_–C_4_	4.86	Z-X-M-Γ-Z, M-X
		V_3_–C_4_	4.97	Z-X-M, Z_1_-M-X-P
		V_3_–C_5_	5.17	Z-X-M, Z_1_-M-X-P
		V_4_–C_4_	4.87	Z-X-M, Z-P-N, Z_1_-M-X-P
		V_5_–C_5_	4.95	Z-X-M, Z_1_-M-X-P
S_5_	5.43	V_1_–C_6_	5.10	Z-X-M, P-N-Z_1_-M, X-P
		V_4_–C_6_	5.33	Z-X-M-Γ-Z, M-X
		V_5_–C_2_	5.87	Z-X-M, P-N-Z_1_-M, X-P
S_6_	6.16	V_6_–C_4_	6.05, 6.18	Z-X-M, Z_1_-M-X-P
		V_6_–C_3_	6.43	Z-X, Z_1_-M-X-P
		V_9_–C_3_	6.22	Z-X-M, Z_1_-M-X-P
		V_9_–C_1_	6.55	Z-X-M, P-N, X-P
S_7_	6.84	V_11_–C_3_	6.72	Z-X-M, P-N, Z_1_-M
		V_9_–C_5_	6.85	Z-X, P-N, Z_1_-M
		V_9_–C_7_	6.94	Z-X-M, P-N, Z_1_-M

Location of the corresponding wave vectors (k) in the BZ for I-42 m Tl_2_HgGeSe_4_ are presented. We denote the levels as (V_1_, V_2_, V_3_,…Vi) up to down from the top of valence band, (C_1_, C_2_, C_3_,….C_i_) down to up from the bottom of conduction band. Structures location and subpeaks position are given in eV.

**Table 9:** Peak locations of the ${\mathcal{E}}_{2}^{\parallel c}(\omega )$ spectrum with the dominant interband transition (IT)_s_ contributions to the considered structure (S_i_) and location of the corresponding wave vectors (k) in the BZ for I-42 m Tl_2_HgGeSe_4_ are presented.

Structure	Location	Band to band transition	Sub-peak position	Brillouin zone region
S_1_	2.73	V_1_–C_1_	2.57	Z-X-M, P-N-Z_1_, M-X-P
		V_3_–C_1_	2.73	Z-X-M, P-N-Z_1_, M-X-P
		V_3_–C_2_	2.61	Z-X, M-Γ-Z, Z_1_-M-X-P
		V_1_–C_4_	2.58	Z-X, M-Γ-Z, Z_1_-M, X-P
S_2_	3.90	V_1_–C_2_	3.79	Z-X-M, P-N-Z_1_-M-X-P
		V_1_–C_3_	3.34	Z-X-M, N-Z_1_-M-X-P
		V_2_–C_2_	3.15	Z-X-M, P-N-Z_1_-M, X-P
		V_3_–C_1_	3.14	Z-X-M, N-Z_1_, M-X-P
		V_4_–C_1_	3.20	Z-X-M, N-Z_1_-M, X-P
S_3_	4.42	V_1_–C_3_	4.13	Z-X-M, X-P
		V_2_–C_3_	4.28	Z-X-M, Z_1_-M-X-P
		V_3_–C_2_	4.16	Z-X-M, N-Z_1_, M-X-P
		V_4_–C_2_	4.02	Z-X-M, Z_1_-M-X-P
		V_8_–C_1_	4.31	Z-X-M, N-Z_1_, M-X-P
S_4_	5.56	V_1_–C_4_	5.11	Z-X, M-Γ, N-Z_1_-M-X-P
		V_1_–C_5_	5.63, 5.72	Z-X, M-Γ-Z, Z_1_-M-X-P
		V_1_–C_6_	5.78	Z-X, M-Γ, M-X-P
		V_2_–C_4_	5.80	Z-X, M-Γ, Z_1_-M-X-P
		V_3_–C_3_	5.84	Z-X, M-Γ-Z, Z_1_-M-X-P
		V_3_–C_4_	5.91	Z-X, M-Γ, M-X-P
S_5_	6.27	V_1_–C_6_	6.23	Z-X, M-Γ, Z_1_-M, X-P
		V_1_–C_7_	6.37	Z-X, M-Γ-Z, Z_1_-M-X-P
		V_1_–C_8_	6.52	Z-X-M-Γ, Z_1_-M-X-P
		V_2_–C_5_	6.61	Z-X, N-Z_1_-M, X-P
S_6_	7.22	V_9_–C_3_	7.13	Z-X, M-Γ, Z_1_-M-X-P
		V_8_–C_3_	7.46	Z-X, M-Γ, Z_1_-M-X
		V_8_–C_4_	7.51	Z-X, M-Γ, Z_1_-M

We denote the levels as (V_1_, V_2_, V_3_,…V_i_) up to down from the top of the valence band, (C_1_, C_2_, C_3_,…C_i_) down to up from the bottom of the conduction band. Structures location and subpeaks position are given in eV.

### XPES experiments: recorded spectrum of as grown crystal (the contaminated surface)

**Figure 12:**
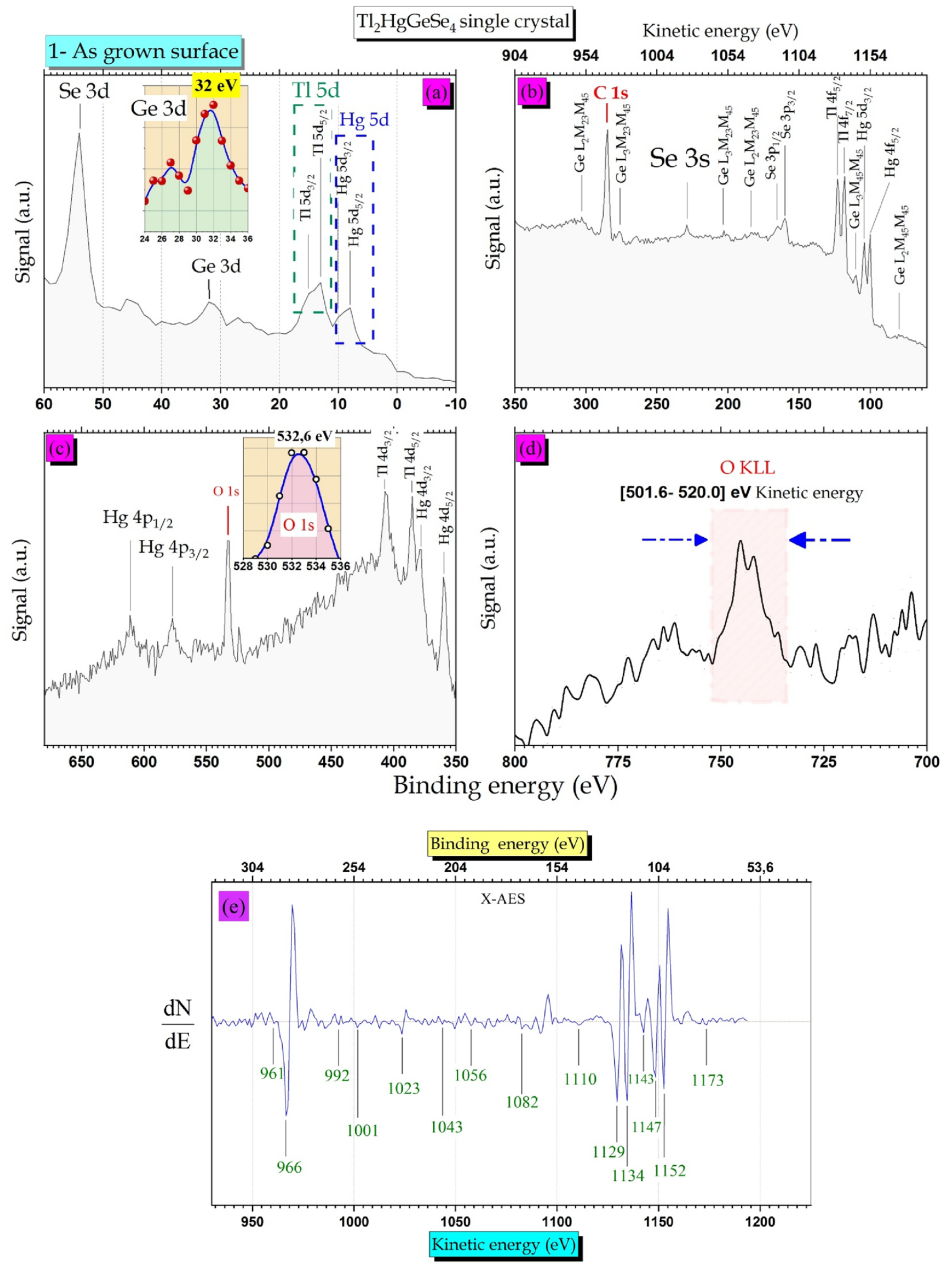
The XPES inner shells spectra of I-42 m Tl_2_HgGeSe_4_ as-synthesized crystal. (**a**) 5d for (Tl and Hg), 3d for (Se and Ge) and the zoomed view of Ge 3d photoelectron. (**b**) C 1 s, Auger electrons of germanium (Ge LMM lines) induced by X-ray beam and 4f. for (Tl and Hg) (**c**) O 1 s, Hg for (4p and 4d), Tl 4d and zoomed view of O 1 s. (**d**) O KLL Auger line and (**e**) the derivative mode (dN(E)/dE) of range (2) which contain major of germanium Auger lines induced by X-Ray excitation (Mg anode, K_alpha_ = 1.2536 keV).

The cruciality of spectroscopic characterizations (e.g. XPES and RS) in nanoscience, medicine, aerospace, communication, manufacturing and many more fields is well known. This part is devoted to gaining further elucidations into how to interpret the Raman vibration modes. In this context, XPES measurement is necessary to verify the elemental composition of the Tl_2_HgGeSe_4_ as-grown sample which was the same surface subjected to Raman spectroscopy tests. We introduce the prepared samples with dimensions of about 1 $\times$ 0.7 $\times$ 0.2 cm^3^ to the analysis chamber under a vacuum better than 8⋅10^–10^ mbar. The X-Ray beam was produced with Mg anode with K_alpha_ = 1.2536 keV. The interaction spot was about 4 mm^2^ area. It is worth knowing that the excited depth using X-Ray photon is more important than the other excited by Lasers. As shown in Fig. 12a–d), we divide the recorded survey spectra into four energy ranges, aiming to clarify more about the as-grown surface response. The core-level peaks related to (Tl and Hg)-(4d, 4f and 5d), (Ge and Se)-3d, Se-(3p and 3 s), Hg-4p, (Ge LMM and O KLL) Auger lines, O 1 s and C 1 s are well distinguished. The Ge LMM auger lines are more localized in the energy range from 60 till 340 eV of binding energy (910 till 1190 eV) kinetic energy, Fig. 12b. The signals stand for core level spectra of carbon (C 1 s peak centred at 284.6 eV) and oxygen (O 1 s peak centred at 532.6 eV) viewing sharp, narrow and intense (see Fig. 12b and c)**.**

A Ge 3d photoelectron signal is localized in the intermediate region between Tl 5d and Se 3d peaks with E_Bin_ = 32.0 eV and a small satellite centred at 27.0 eV, see Fig. 12a and the enlarged shape. Furthermore, Fig. 12d presents the oxygen O KLL Auger line represented in a wide peak centred with kinetic energy between [501.6–520] eV. The Ge LMM Auger lines spectra are also presented in the derivative mode (Fig. 12e) to follow peak features of as-grown crystal and themes energy shifts compared to other experimental findings. Wherefore, the later records were meaningful and led us to establish further elucidations into the oxidized states that could be present in our crystal.

#### Selenium and germanium oxidized states

It is well known that samples undergone to an exposition of laboratory air for more than six hours (6 h) which is considered as an ex-situ oxidations process^62–66^ affecting mainly at the chemical composition of the crystal under investigation. Hellgren et al.^64^ reveal that the ZnSe sample etched by NH_4_OH:C_2_H_6_O solution has a huge contribution of Se/SeOH, being in excellent correlation with oxygen O 1 s signal which is localized at 532.9 eV binding energy. This is in good conformity with our finding (532.6 eV) with a deviation that doesn’t exceed 0.3 eV. Certainly, our results suggest that the presence of the hydroxyl group –(OH) at the Tl_2_HgGeSe_4_ crystal surface originates links with selenium being in the specimen to form Se–OH bonds. These results are also well confirmed by the following Raman spectroscopy. On the other side, the Ge Auger peaks as shown in both Fig. 12b and e themes amplitudes in a derivative mode (dN(E)/dE), help us to gain further information. Wang et al.^65^ report in detail the energetic shifts in Ge Auger lines and the new Auger transition happens by comparing results obtained from pure germanium and others from stable germanium oxide (GeO_2_). The Auger electron spectroscopy (AES) results display that the Ge LMM line of pure germanium is more susceptible to the oxidation phenomenon. There are shifts in Auger peaks toward low energies from pure to oxidized state. Our results show strong Auger lines induced from X-Ray excitation as follows: Ge L_2_M_23_M_45_ (966 eV), Ge L_3_M_23_M_45_ (1056 eV), Ge L_3_M_23_M_45_ (1073 eV), Ge L_3_M_45_M_45_ (1143 eV), Ge L_2_M_45_M_45_ (1182 eV) and also O KL_23_L_23_(508.6 eV) kinetic energy being in excellent agreement with results extracted from AES spectroscopy of good and stable germanium dioxide (GeO_2_)^64^.

These results confirm also the formation of germanium oxide at the surface of the as grown Tl_2_HgGeSe_4_ crystal. Prabhakaran et al.^62^ reported that using both XPES measurements and He I Ultraviolet Photoelectron Spectroscopy (He I UPS), the localization of the Ge 3d line related to pure Ge(111) exposed 6 h to laboratory air is characterized by energy bond of E_Bin_ = 32.0 eV attributed to the formation of new oxide (GeO). Compared to the Ge 3d peak in our study, it shapes with similar features suggesting the formation of small surface species of this oxide. Other interest observation focused on the oxygen photoelectron O 1 s line which centered at 532.4 eV, is attributed mainly to the GeO_2_ formation with covalent bonds nature of Ge–O–Ge type. This peculiarity is fairly consistence with our results with a deviation of 0.2 eV.

#### Valence band X-ray photoelectron spectroscopy (VB-XPES) after UHV cleaning and predictive Total DOS curves: comparison study

**Figure 13:**
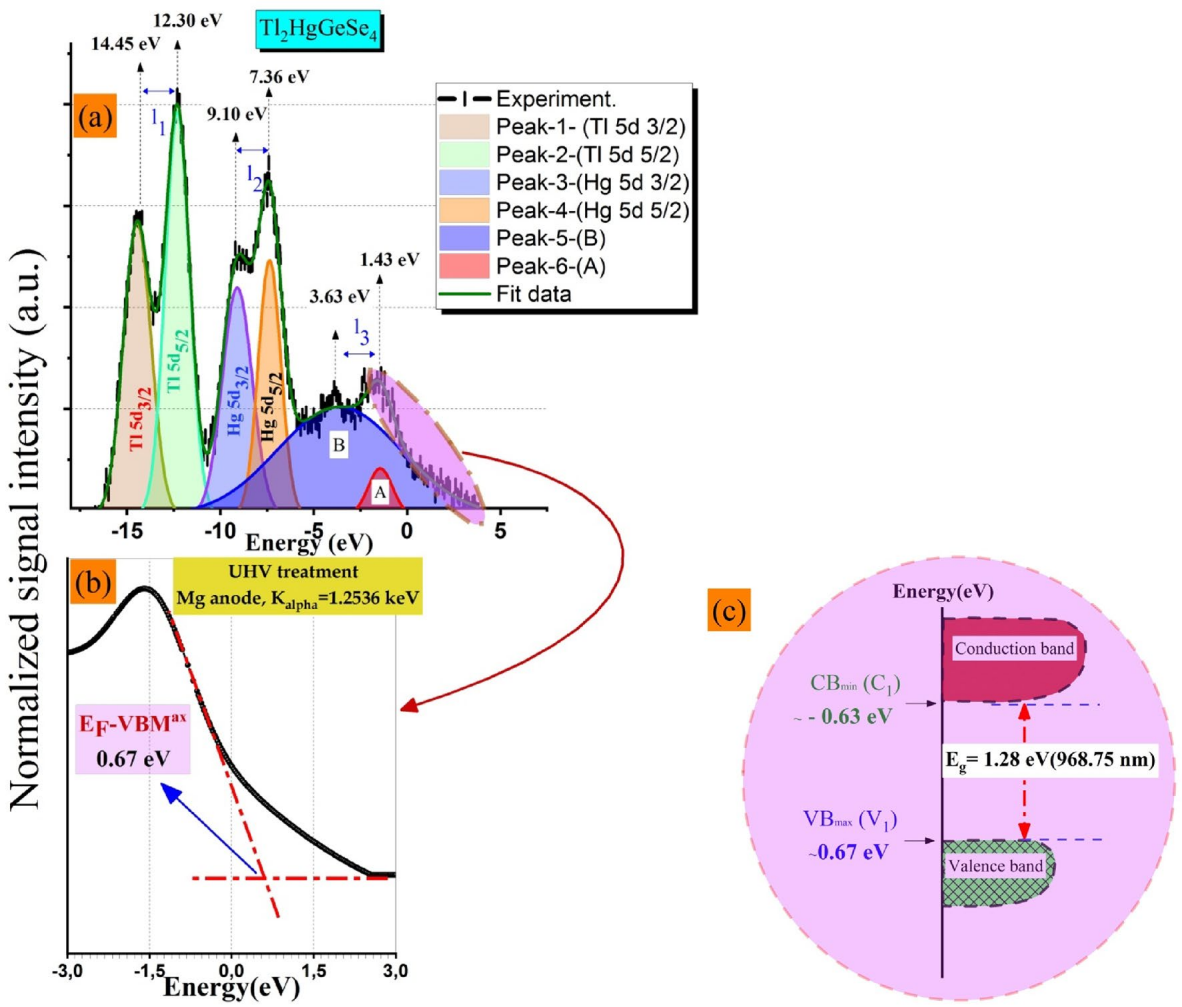
(**a**) Experimental valence band X-ray photoelectron spectroscopy (VB-XPS) of Tl_2_HgGeSe_4_ compound. A Gaussian deconvolution combined with Shirley background subtraction was performed at the experimental VB-XPS raw data enable us to detect four well pronounced peaks (Peak 1, 2, 3 and 4) and others (A and B) with low intensities. See text for more details. (**b**) The valence band (VB-XPES) shows E_F_-E_VBM_ = (0.67 $\pm$ 0.05) eV and (**c**) Schematic illustration for the electronic band structure of Tl_2_HgGeSe_4_ compound.

It is of great importance to present an accurate result relevant to the upper valence band region for such a compound. In fact, there is a lack of a database related to quaternary like setting VBM^ax^ and its energy shifts. This former can help researchers who consider as an important milestone to follow easily homogeneity, stoichiometry and control surface features if it is subjected to various physical treatments or chemical processes (e.g., elemental doping, molecular epitaxy, UHV annealing, coating and so on). We show in Fig. 13a the experimental valence band picked up from X-Ray PhotoElectron Spectroscopy (VB-XPES) of the cleaning state of the Tl_2_HgGeSe_4_ crystal. It should be noted that the total disappearance of oxygen O 1 s signal from the survey spectrum (not shown here) caused a substantial increase in the intensity of the main peaks which reached about 8%. Figure 13a also shows a Gaussian fitting of the valence band (VB-XPES) spectrum after Shirley background subtraction. There are four sharp intense peaks (Tl 5d_3/2_ at 14.45 eV, Tl 5d_5/2_ at 12.30 eV, Hg 5d_3/2_ at 9.10 eV and Hg 5d_5/2_ at 7.36 eV) and two others with low intensity (A at 3.63 eV and B at 1.43 eV), respectively. The interest peak features such as localization, full width in half maximum (FWHM), peak to peak distance (I_i_), peak area and intensity are given in Table 10. We also listed in Table 10 some results related to the present full potential theoretical part, for comparison.

**Table 10:** Gaussian fitting of experimental valence band (VB-XPES) spectrum for UHV treated Tl_2_HgGeSe_4_ samples and some results extracted from TDOS using (TB-mBJ and new KTB-mBJ).

	Peak-1-Tl **5d**_**3/2**_			Peak-2-Tl **5d**_**5/2**_			Peak-3-Hg **5d**_**3/2**_			Peak-4-Hg **5d**_**5/2**_			Peak-5-(B)	Peak-6-(A)
	Expt	TB	nKTB	Expt	TB	nKTB	Expt	TB	nKTB	Expt	TB	nKTB	Expt	
Energy(eV)	14.45	13.70	13.81	12.30	12.59	12.53	9.10	7.77	8.17	7.36	6.90	7.63	3.63	1.43
Peak to peak distance (eV)	I_1_						I_2_						I_3_	
	2.15		1.11		1.28		1.74		0.87		0.54		2.2	
FWHM (eV)	1.74			1.55			1.88			1.43			7.76	1.52
Peak area (a.u.)^2^	532.94			655.03			447.70			380.97			881.33	74.81
Intensity (a.u.)	28.85	4.00	4.08	40.05	9.71	12.41	25.83	5.02	8.18	34.26	15.44	8.06	12.06	13.43

Employing a linear extrapolation performed at the fitted valence band edge as depicted in Fig. 13b, it seems clear that the E_F_-VBM^ax^ value was deduced to be (0*.*67 ± 0*.*05) eV for tetragonal Tl_2_HgGeSe_4_ clean surface. This result enables us to determine experimentally for the first time the band gap diagram of Tl_2_HgGeSe_4_ single crystal as shown in Fig. 13c.

**Figure 14:**
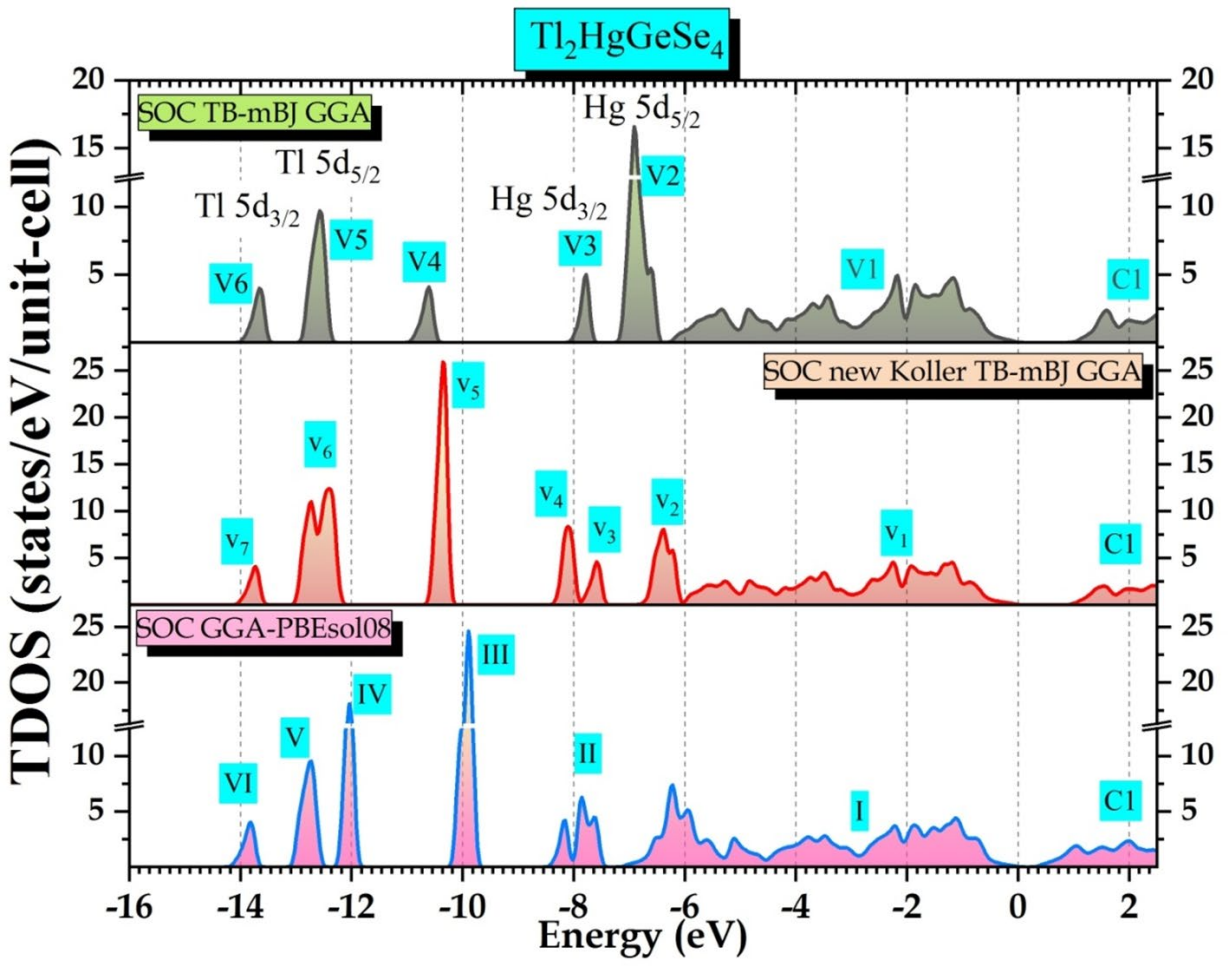
The predictive total densities of states (TDOS) spectra of Tl_2_HgGeSe_4_, calculated within TB-mBJ, new Koller TB-mBJ and GGA-PBEsol08 models.

In other side, we aspire as far as possible to validate our predictive total DOS spectra with VB-XPES counterparts. Figure 14 gathers these three predictive results.

We could divide TDOS related to SOC TB-mBJ functional into six energy ranges, a wide structure V1 with a barycenter of 3.17 eV and other narrow peaks V2, V3, V4, V5, and V6. As we go to the new KTB-mBJ potential TDOS spectrum, V2 peak (6.90 eV) decreases in intensity and merges somewhat in V1, V3 (7.78 eV) splits into two peaks V_3_ (7.58 eV) and V_4_(8.10 eV), V4 (10.60 eV) shifts toward low energies and increase significantly in intensity presented in V_5_ (10.35 eV), V5 (12.56 eV) changes to a wide structure with two well distinguished tops (Structure V_6_ with tops of 12.40 eV and 12.72 eV) and the V6 peak (13.70 eV) is still approximately similar in shape and intensity as V_7_. When going further to GGA-PBEsol potential, V_2_ merges totally in V_1_ forming a new large structure I, peak V_4_ superposes with peak V_3_ and makes them increase in intensity where the sum forms a structure II, V_5_ decreases in intensity and also shifted toward low energies as peak III, V_6_ structure splits into two well pronounced peaks (V and IV) with a pseudo gap of 0.13 eV and V_7_ peak shifts slightly toward higher energies. Finally, the bottom level of the conduction band (BCB) widens relatively with the sequence TB-mBJ$\to$ new KTB-mBJ$\to$ GGA-PBEsol 08. We focus in this context on the closest calculation to the experimental measurements. Tb-mBJ potential shows this tendency. We note that the upper levels of the valence band (UVB) denoted V1 between [− 5.25 eV to 0 eV] corresponds to a relative hybridization occurring between electrons of Tl-s and Se-p orbitals. Other peaks shown (V2 and V3) are predominantly assigned to Hg (5d_5/2_ and 5d_3/2_) orbital contribution, respectively. The peak denoted V4 arises between [− 11.04 eV to − 10.36 eV] and it is attributed to a moderate participation of Ge–s electronic states with Se-s electronic states. Other peaks so far located at 13.70 eV and 12.59 eV, are attributed to a huge contribution of Tl (5d_3/2_ and 5d_5/2_), respectively. Respecting the VB-XPES results, Hg (5d_3/2_ and 5d_5/2_) and Tl (5d_3/2_ and 5d_5/2_) electronic states positions, the peaks detected by calculations within TB-mBJ potential fairly agree with the experiment. The slight deviations (− 0.75 eV and + 0.29 eV) and (− 1.33 eV and − 0.46 eV), respectively, may be attributed to the fact of the Hubbard amendment which was not introduced in our findings compared to others^1^.

Interestingly, the peak intensity has an important physical meaning, related to which electronic state it contributes more than the other in such a response. Our results show the closest concordance compared to VB-XPES cleaning state results with unambiguous ratios $\frac{I({Tl 5d}_{ 5/2})}{I({Tl 5d }_{3/2})}$ and $\frac{I({Hg 5d}_{ 5/2})}{I({Hg 5d }_{3/2})}$ greater than unity ($>1$), conversely to what recently reported^1^.

### Raman Spectra: experiment and theory

**Figure 15:**
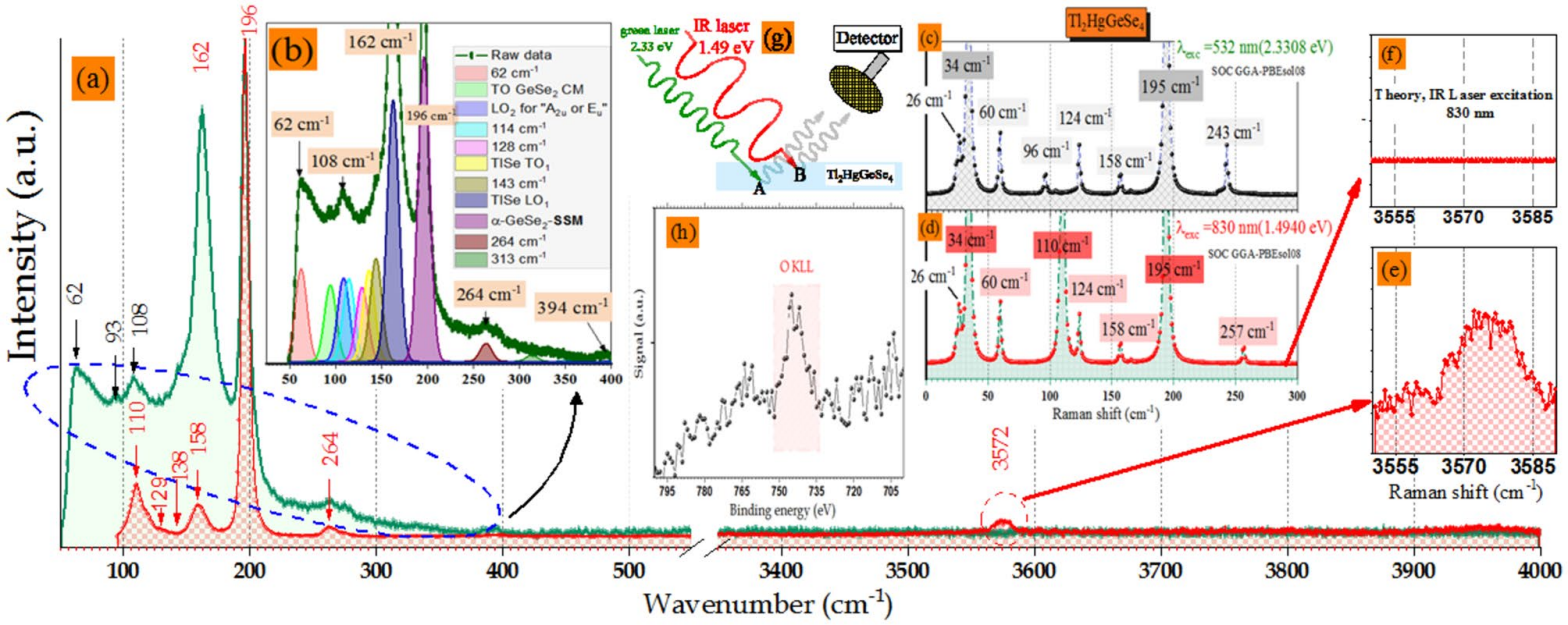
(**a**) Survey Raman spectra [50–4000] cm^–1^ with green (532 nm) and [90–4000] cm^–1^ with infrared (830 nm) laser excitation. (**b**) Gaussian deconvolution was performed at 60 to 400 cm^-1^ Raman shift (green laser excitation). (**c**) and (**d**) The predicted Ramen spectra of Tl_2_HgGeSe_4_ crystal at ambient pressure calculated with Pseudo Potential Plane Wave method (PP-PW) as implemented in Castep code, source excitation of 2.3308 eV and 1.4940 eV, respectively. (**e**) The correspondent vibration of –(OH) groups (**f**) the computed contourpart. (**g**) A schematic illustration of laser-matter interaction with A and B different depths and (**h**) The O KLL auger line localized between [521.6–520.0] eV kinetic energy.

The present Raman spectroscopy (RS) characterization was complimentary to XPES measurements. RS is a powerful, qualitative and non-destructive technique. Figure 15a shows survey spectra of the Raman test using two different laser excitations: green for deeper states (**A**) and IR for shallow layers (**B**) (see Fig. 15g**)**.

We draw the reader’s attention that the initial states of the samples introduced to both Raman and XPES equipments were the same. Using laser excitation of 532 nm (2.3308 eV), the recorded spectrum at Room Temperature (RT) has much more peculiarities than the other recorded using IR 830 nm (1.494 eV). In order to gain more insights for Tl_2_HgGeSe_4_ single crystal vibration mode, we have made a Gaussian fit of the RS spectrum (Fig. 15b) after intensity normalization and background subtraction. The sub-peak assignments were well discussed by Vu et al.^1^ and references therein. However, using the previous details on the XPES results, section "XPES experiments: recorded spectrum of as grown crystal (the contaminated surface)", some vibration modes should be more clarified. Fraysse et al.^67^ have reported that based on both experimental and theoretical Raman spectroscopy data, the alpha-quartz-type GeO_2_ single crystals have a mode localized at a wavenumber of 264 cm^–1^ with FWHM equal to 10 cm^–1^, similar to that as in the present work, attributed to “O–Ge–O” twisting labeled as A1 (TO2) line. This result is in good agreement with our previous work (checked by XPES) and others with (**He I** UPS analysis). Besides, this vibration mode A1(TO2) appears obviously when we use two different excitation sources (well-pronounced by IR excitation) that means a relative oxygen diffusing in the bulk of the as-grown Tl_2_HgGeSe_4_ crystal. In other hand, with the help of the pseudo potential plane wave calculations (PP-PW), we run a predictive Raman spectroscopy to complete the lack in experimental Raman records which was due to the limitation of equipment detection. The predictive Raman spectra presented in Fig. 15c and d are made by convolution of the computed intensities with an appropriate Lorentzian function. The resolution spectra presented in Fig. 15c and d is ca. 5 cm^–1^. There are no remarked signals starting from 300 till to 4000 cm^–1^ using both 532 nm and 830 nm wavelengths excitations. The present theoretical Raman spectra of tetragonal Tl_2_HgGeSe_4_ show the vibration modes in its perfect crystallization (no impurities, dislocations, antisites…etc.). In each spectrum, there are eight pronounced lines. The vibration modes at 26, 34, 60, 124, 158 and 195 cm^–1^ are detected in the both theoretical spectra. When we decrease the excitation energy from 2.33 eV to 1.49 eV, the peaks corresponding to frequencies equal to 96 cm^–1^ and 243 cm^–1^ disappear completely. Two new peaks (sharp intense at 110 cm^–1^ and slightly pronounced fine-structure peculiarity at 257 cm^–1^) arise. Lowndes et al.^68^ report that the modes at 26, 34 and 60 cm^–1^ are attributed to pure thallium optic modes with uncertainty of $\pm$ 1 cm^–1^.

Combining by the present results, a fine correlation between the theoretical and experimental data was established. So, based on data presented in Fig. 15e, f and h and using IR laser source, we certainly can attribute the peak with wavenumber of 3572 cm^–1^ to Se–OH vibrations (checked by XPES and no signal provided from DFPT prediction), dominantly localized at the shallow layers of our crystal (comparing to the green excitation one).

## Conclusions

Theoretical studies have been performed in the present work to explore thermodynamic properties, structural and dynamic stabilities, dependence of unit-cell parameters and elastic constants upon hydrostatic pressure, charge carrier effective masses, electronic and optical properties, contributions of interband transitions to the Brillouin zone of the Tl_2_HgGeSe_4_ crystal synthesized recently in Ref.^1^. The theoretical methods used in the present work are based on the density-functional perturbation theory (DFPT). To verify the correctness of the present theoretical results, we treat different models for XC potential, in particular TB-mBJ, new KTB-mBJ, and GGA-PBEsol08. It has been established that, with respect to the energy band gap of the Tl_2_HgGeSe_4_ crystal, the smallest deviation with the experimental data we receive when the calculations are carried out via the TB-mBJ approach, followed by calculations within new KTB-mBJ and, then, within GGA-PBEsol08 XC potentials. In particular, all the pronounced peaks of the phonon DOS curve are located at positive frequencies ranging from 0.36 to 8.34 THz, confirming the dynamical stability of the titled crystal. Further, when we consider the influence of the hydrostatic pressure in the present calculations, we find that an increase of the applied pressure from 0 to 20 GPa, the ratio c/c_0_ decreases more slowly than a/a_0_ . This trend indicates that the Tl_2_HgGeSe_4_ crystal is much more compressible along the a-axis than along the c-axis. This result is consistent with the calculations of elastic sound wave velocities indicating that the Tl_2_HgGeSe_4_ crystal has a higher velocity along the longitudinal c direction [001] (v_L_[001] = 3044 m.s^–1^). The charge effective mass values of electron and hole, $m_{e}^{{^{*} }}$ and $m_{h}^{{^{*} }}$, vary essentially when the direction in high symmetry changes indicating a relative anisotropy of charge-carriers mobility in Tl_2_HgGeSe_4_. Furthermore, the present theoretical results show that the Young modulus and compressibility are characterized by the maximum and minimum values ($E^{max}$ and $E^{\min }$) and ($\beta^{max}$ and $\beta^{\min }$) that equal to (62.032 and 28.812) GPa and (13.672 and 6.7175) TPa^–1^, respectively, indicating the presence of elastic anisotropy in the Tl_2_HgGeSe_4_ crystal. The calculated hole effective masses are lighter than those of electrons indicating the p-type electroconductivity of the Tl_2_HgGeSe_4_ crystal confirming the experimental findings^1^. In the latter work it was concluded that the VB topmost point occurs at Z point; however, the present data indicate that the VBM^ax^ possesses two wide tops at almost equivalent energy levels, in fact, at the M and Z points with a difference reaching about tenths of meV between their tops. Nevertheless, the conclusion of Ref.^1^ regarding the non-direct bandgap nature in the Tl_2_HgGeSe_4_ crystal is confirmed by the present calculations.

The present DFT calculations reveal that, the Tl-Se bonds in Tl_2_HgGeSe_4_ are ionic, while the total ionic radii (d_*R*(Hg2+)+*R*(Se2-)_ and d_*R*(Ge_^4+^_)+__*R*(Se_^2–^_)_) are larger than the experimental ones indicating the predominant covalent nature for both Hg-Se and Ge-Se bonds in this compound. Following the fact that the electronegativity difference (ED) is a helpful factor to measure the stiffness of the chemical bonds and the highest ED value corresponds to the weak ones. In the case of the crystal under consideration we observe the following sequence: ${\Delta\upchi }_{\text{Ge}-\text{Se}}=0.54<{\Delta\upchi }_{\text{Hg}-\text{Se}}=0.55<{\Delta\upchi }_{\text{Tl}-\text{Se}}=0.93$. So, the strongest bonds were formed between Se and Ge species to form hard [GeSe_4_] blocks. The combination between two complemantary techniques (XPES and RS), suggest that the presence of the hydroxyl group –(OH) at the pristine Tl_2_HgGeSe_4_ crystal surface originates binding with selenium being present in the top surface layers of the crystal to form Se–OH bonds. The present calculations of the Raman spectra using a red excitation 830 nm (1.49 eV) reveal a good correspondence with the experimental spectra of the Tl_2_HgGeSe_4_ crystal measured at room temperature.

## Data Availability

The data is available within the manuscript.
