# Author Correction: Structural, elastic, electronic, optical and anisotropy properties of newly quaternary Tl2HgGeSe4 via DFPT predictions associated to XPES and RS experiments

**DOI:** 10.1038/s41598-024-77722-x

**Published:** 2024-11-13

**Authors:** Mohamed Salah Halati, Oleg Yu Khyzhun, Abderrazak Khireddine, Michal Piasecki, Ilona Radkowska, Khaled Hamdi Cherif, Zakia Lounis, Yves Caudano, Abdelhak Bedjaoui, Ahmad Alghamdi, Prabhu Paramasivam, Chander Prakash, Sherif S. M. Ghoneim

**Affiliations:** 1 Scientific and Technical Research Centre for Arid Areas (C.R.S.T.R.A), Water Resources and Treatments Team, Omar El Barnaoui, Campus of Mohamed, Khider University of Biskra, 07000 Biskra, Algeria; 2 Laboratory of Materials (LabMat), National Polytechnique School (ENP-Maurice Audin -Oran-), El M’naouer, P.O. Box 1523, 31000 Oran, Algeria; 3 Modeling and Simulations for Laser Applications Team, Research Center in Industrial Technologies (CRTI), P. O. Box 64, 16014 Cheraga, Algiers Algeria; 4 Frantsevych Institute for Problems of Materials Science, National Academy of Sciences of Ukraine, 3 Krzhyzhanivsky Street, Kyiv, 03142 Ukraine; 5 Department of Experimental Physics and Information‑Measuring Technology, Lesya Ukrainka Volyn National University, 13 Voli Avenue, Lutsk, UA‑43025 Ukraine; 6 Laboratory for Developing New Materials and Their Characterizations, Department of Physics, Faculty of Science, University Ferhat Abbas Setif 1, 19000 Setif, Algeria; 7 Jan Dlugosz University in Częstochowa, Armii Krajowej 13/15, PL‑42‑217 Częstochowa, Poland; 8 Research Unit Lasers and Spectroscopies (UR‑LLS), naXys & NISM, Université de Namur, Rue de Bruxelles 61, 5000 Namur, Belgium; 9 Department of Technology, Faculty of Technology, Bejaia University, 6000 Bejaia, Algeria; 10 Department of Mechanical and Industrial Engineering, College of Engineering and Computing in Al-Qunfudhah, Umm Al-Qura University, Mecca, Saudi Arabia; 11 Centre of Research Impact and Outcome, Chitkara University, Rajpura, Punjab 140401 India; 12 Department of Mechanical Engineering, Mattu University, 318 Mettu, Ethiopia; 13 University Centre for Research and Development, Chandigarh University, Mohali, Punjab 140413 India; 14 Department of Electrical Engineering, College of Engineering, Taif University, P.O. BOX 11099, 21944 Taif, Saudi Arabia

Correction to: *Scientific Reports* 10.1038/s41598-024-67231-2, published online 15 July 2024

The original version of this Article contained an error in the spelling of the author Ahmad Alghamdi which was incorrectly given as Ahmed Alghamdi.

The original Article has been corrected.

